# A *DOCK1* Gene-Derived Circular RNA Is Highly Expressed in Luminal Mammary Tumours and Is Involved in the Epithelial Differentiation, Growth, and Motility of Breast Cancer Cells

**DOI:** 10.3390/cancers13215325

**Published:** 2021-10-23

**Authors:** Mami Kurosaki, Mineko Terao, Dawei Liu, Adriana Zanetti, Luca Guarrera, Marco Bolis, Maurizio Gianni’, Gabriela Paroni, Gregory J. Goodall, Enrico Garattini

**Affiliations:** 1 Laboratory of Molecular Biology, Istituto di Ricerche Farmacologiche Mario Negri IRCCS, 20156 Milano, Italy; mami.kurosaki@marionegri.it (M.K.); mineko.terao@marionegri.it (M.T.); adriana.zanetti@marionegri.it (A.Z.); luca.guarrera@marionegri.it (L.G.); marco.bolis@marionegri.it (M.B.); maurizio.gianni@marionegri.it (M.G.); gabriela.paroni@marionegri.it (G.P.); 2 Centre for Cancer Biology, An Alliance of SA Pathology and University of South Australia, Adelaide, SA 5000, Australia; Dawei.Liu@unisa.au (D.L.); Greg.Goodall@unisa.au (G.J.G.); 3 Institute of Oncology Research, USI, University of Southern Switzerland, 6500 Bellinzona, Switzerland; 4 Department of Medicine, The University of Adelaide, Adelaide, SA 5005, Australia

**Keywords:** circular RNAs, breast cancer, RNA sequencing, differentiation, epithelial-to-mesenchymal transition (EMT)

## Abstract

**Simple Summary:**

In the present study, we define the expression of 15 selected circular RNAs, using a panel of 18 breast cancer cell lines recapitulating the heterogeneity of tumors and consisting of three distinct groups according to the mesenchymal/epithelial phenotype. We identify *circDOCK1-1* generated from the *DOCK1* gene as an important regulator of the epithelial differentiation of mammary tumour cells. In addition, our high-throughput transcriptomic studies provide information on the genes that are regulated by *circDOCK1-1*, casting new light on the gene networks involved in the processes underlying the development of mammary tumours. Our results further indicate that *circDOCK1-1* controls cell–cell adhesion and consequently random as well as directional motility. Besides their interest from a basic point of view, the data generated may be of translational relevance, as they suggest that strategies aimed at increasing the amounts or at stimulating the activity of *circDOCK1-1* may be of significance for the treatment of a subset of triple-negative breast cancer patients.

**Abstract:**

Circular RNAs are regulatory molecules involved in numerous cellular processes and may be involved in tumour growth and diffusion. Here, we define the expression of 15 selected circular RNAs, which may control the process of epithelial-to-mesenchymal transition, using a panel of 18 breast cancer cell lines recapitulating the heterogeneity of these tumours and consisting of three groups according to the mesenchymal/epithelial phenotype. A circular RNA from the *DOCK1* gene (*hsa_circ_0020397*) shows low/undetectable levels in triple-negative mesenchymal cell lines, while its content is high in epithelial cell lines, independent of estrogen receptor or HER2 positivity. RNA-sequencing experiments performed on the triple-negative/mesenchymal *MDA-MB-231* and *MDA-MB-157* cell lines engineered to overexpress *hsa_circ_0020397* demonstrate that the circRNA influences the expression of 110 common genes. Pathway analysis of these genes indicates that overexpression of the circular RNA differentiates the two mesenchymal cell lines along the epithelial pathway and increases cell-to-cell adhesion. This is accompanied by growth inhibition and a reduction in the random/directional motility of the cell lines. The upregulated *AGR2*, *ENPP1*, and *PPP1R9A* genes as well as the downregulated *APOE*, *AQP3*, *CD99L2*, and *IGFBP4* genes show an opposite regulation by *hsa_circ_0020397* silencing in luminal *CAMA1* cells. The results provide novel insights into the role played by specific circular RNAs in the generation/progression of breast cancer.

## 1. Introduction

Circular RNAs are covalent circles of single-stranded RNA that are generated from non-canonical back-splicing of pre-mRNAs and non-coding RNAs [1,2,3]. Circular RNAs are a largely overlooked class of regulatory molecules, as they are difficult to identify and quantitate with the traditional methods used for the analysis of RNA-sequencing (RNA-seq) data [4]. Nevertheless, recent advances in the field indicate that circular RNAs may be involved in numerous biological processes and are likely to be key regulators of multiple aspects of the cell and tissue homeostasis [5]. In particular, circular RNAs are likely to play a significant role in the homeostasis of the tumour cell [6,7], since they are involved in the control of cell cycle [8], tumorigenesis, and chemoresistance [9,10]. This is predominantly the consequence of the fact that circular RNAs modulate the activity of regulatory proteins and RNAs, such as transcription factors [11] and microRNAs [12,13,14]. Indeed, there is emerging evidence indicating that one of the main mechanisms underlying the regulatory action of circular RNAs is related to the ability of these molecules to act as sponges and to sequester microRNAs [15]. Despite this background, there are relatively few studies focussing on the involvement of circular RNAs in the development and progression of solid tumours, with particular reference to breast cancer [15,16,17], a very heterogeneous type of tumour consisting of a large collection of different diseases [18,19,20,21].

In the present study, we focus our interest on a small set of circular RNAs that may be involved in the process of epithelial-to-mesenchymal transition (EMT) [22] and we define their profile of expression in a relatively large panel of cell lines recapitulating the heterogeneity of the disease [23]. One of the 126 circular RNAs predicted to derive from the *DOCK1* gene (*hsa_circ_0020397*; *circDOCK1-1* throughout the text) shows the most interesting profile, as it is expressed at low/undetectable levels in triple-negative mesenchymal cell lines, while its content is high in epithelial cell lines. The whole-genome RNA-sequencing (*RNA-seq*) data obtained from two triple-negative mesenchymal cell lines engineered to overexpress *circDOCK1-1* indicate that this circular RNA controls the expression of genes involved in epithelial differentiation and cell adhesion. Among these genes, the upregulated *AGR2*, *ENPP1*, and *PPP1R9A* as well as the downregulated *APOE*, *AQP3*, *CD99L2*, and *IGFBP4* are of particular interest. In fact, the seven abovementioned genes show an opposite behaviours upon *circDOCK1* silencing in luminal *CAMA1* cells. From a functional point of view, forced expression of *circDOCK1-1* in two mesenchymal breast cancer cell lines causes a growth inhibitory effect and a significant reduction in the random/directional motility of both cell lines.

## 2. Materials and Methods

### 2.1. Reagents, Biological, and Computational Resources

All the reagents, the list of the cell lines, and their phenotypic characteristics/source are available in Supplementary Materials. Some of the analyses presented in the study required the use of the TCGA (The Cancer Genome Atlas) dataset of the National Cancer Institute (https://www.cancer.gov/about-nci/organization/ccg/research/structural-genomics/tcga, accessed on 12 May 2021) and the CCLE (Cancer Cell Line Encyclopedia) of the Broad Institute (https://sites.broadinstitute.org/ccle, accessed on 12 May 2021), as indicated in the appropriate sections of the manuscript.

### 2.2. Generation of circDOCK1-1-Overexpressing and Knock-Out Cells

The methodologies used for the generation of the *MDA-MB-231* and *MDA-MB-157* cells characterized by forced expression of *circDOCK1-1* and the *CAMA1* cells genetically engineered for *circDOCK1-1* silencing are described in the Supplementary Methods section of the Supplementary Materials. 

### 2.3. Measurement of the Selected circRNAs and Corresponding Linear mRNAs

Total RNA was extracted using the miRNeasy Mini Kit (Qiagen, Hilden, Germany). The RNA was reverse transcribed using the Maxima H Minus cDNA Synthesis Master Mix (Thermo Fisher Scientific, Waltham, MA, USA) and it was amplified with a Taqman Universal Master Mix (Applied Biosystems, Waltham, MA, USA), using specific Taqman probes (Applied Biosystems). Some of the mRNAs were determined with the SYBR Green PCR Kit (Qiagen) using oligonucleotides as primers. circRNAs were reverse transcribed with the Quantitect Reverse Transcription Kit (Qiagen). The corresponding cDNAs were amplified using the SYBR Green PCR Kit (Qiagen). A list of the Taqman probes and the structure of specific oligonucleotide primers is available in the Supplementary Materials. 

### 2.4. Western Blot Analyses

Whole-cell protein extracts were prepared according to previously described methods [20]. Aliquots of the protein extracts (10 µg) were separated on PAGE and transferred to nitrocellulose membranes. Protein bands were detected with specific antibodies, as detailed in the Supplementary Materials.

### 2.5. Cell Proliferation, Single-Cell Motility, and Wound-Healing Assays

The growth rate of cells was determined with the sulforhodamine B assay. Single-cell motility was examined with a previously described method [24], while wound-healing assays were performed using the method of Jonkman [25].

### 2.6. RNA-Sequencing Studies

For the *RNA-seq* analysis of the *circDOCK1*-overexpressing *MDA-MB-231* and *MDA-MB-157* cells and the respective *pVOID* controls, three biological replicates of each cell line were grown in the presence of G418. For the silencing experiments, three biological replicates of cells were treated for 4 days in the presence/absence of doxycycline. Total RNA was extracted with the miRNeasy Mini Kit (Qiagen). cDNA libraries were prepared with the Illumina TruSeq RNAlibrary kit (Illumina, San Diego, CA, USA) and sequenced on the Illumina NextSeq500 with paired-end 150 base-pair-long reads. The *RNA-seq* data were analysed as described [23] and deposited in the EMBL-EBI Arrayexpress database (Accession No: E-MTAB-10819). The quality of the sequencing data was assessed with the use of FastQC. Sequence alignments to the reference human genome (*GRCh38*) were performed with the “Spliced Transcripts Alignment to a Reference” (*STAR*) software (v.2.5.2a). Gene expression was quantitated with the use of the annotations available in *Gencode* (version 27). Raw counts were processed in the R-statistical environment and differential expression analysis was conducted with the DESeq2 pipeline [26]. Genes endowed with low mean normalized counts were filtered out by the Independent Filtering feature embedded in DESeq2 (alpha = 0.05). Statistical enrichment of gene sets was assessed with the use of the HALLMARK collection.

### 2.7. Statistical Analyses

The details regarding the statistical tests for the analysis of the quantitative data presented are available in the text or figure legends. All the analyses were performed using two-tailed statistical tests. The statistical treatments performed on the *RNA-seq* data are detailed in the above *RNA-Sequencing Studies* paragraph.

## 3. Results

### 3.1. Expression of Selected circRNAs in Breast Cancer Cell Lines 

To identify circRNAs potentially involved in the progression of breast cancer, we previously performed *RNA-seq* studies in *HMLE* (immortalized-human-mammary-epithelial) cells subjected to EMT by exposure to TGFβ (*mesHMLE*) [1]. Relative to the parental *HMLE* counterparts, *mesHMLE* cells showed upregulation of 11 circRNAs and downregulation of 4 circRNAs (Table 1).

We evaluated the expression of the selected circRNAs in 20 breast cancer cell lines recapitulating the heterogeneity of mammary tumours. The panel of cells consisted of three distinct groups, as indicated by principal component analysis (PCA) of the transcriptomic data obtained in each cell line by *RNA-seq* (Table S1 and Figure S1). The *Lum* group contained eight cell lines characterized by a luminal phenotype (Supplementary Materials): (a) oestrogen receptor-negative/progesterone receptor-negative/HER2-positive *SKBR-3*, *HCC-202*, and *HCC-1419* cells; (b) oestrogen receptor-positive/progesterone receptor-negative/HER2-positive *MDA-MB-361* cells; (c) oestrogen receptor-positive/progesterone receptor-negative/HER2-negative *MDA-MB-175VII* and *ZR75.1* cells; and (d) oestrogen receptor-positive/progesterone receptor-positive/HER2-negative *CAMA1* and *HCC-1500* cells. The basal gene expression profiles separated the remaining cell lines, which are characterized by a triple-negative phenotype into two sub-groups, showing an epithelial (6 cell lines; *TNBC*) and a mesenchymal (6 cell lines; *TNBC-mes*) morphology (Supplementary Materials). ssGSEA of the *RNA-seq* data indicated that the gene expression profiles of *TNBC-mes* cells show a general up regulation of the EMT gene networks relative to the *TNBC* counterparts (Figure S2). 

**Figure 1:**
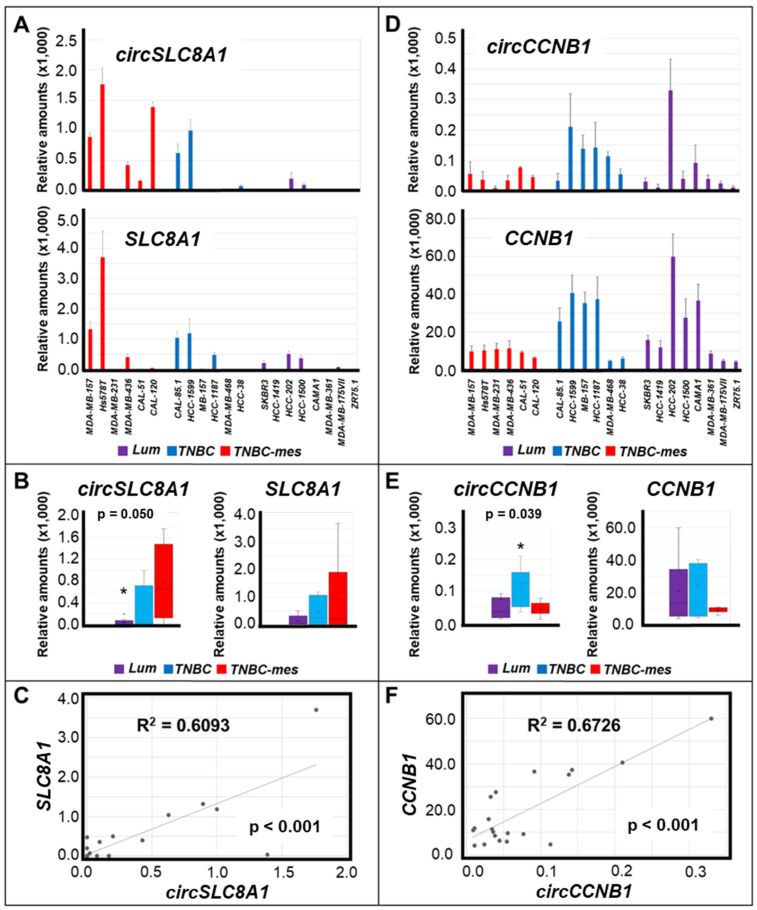
Expression of *circSLC8A1* and *circCCNB1* RNAs as well as the corresponding linear *SLC8A1* and *CCNB1* mRNAs in breast cancer cell lines. (**A**) Total RNA was extracted from the indicated cell lines and the levels of *circSLC8A1* RNA (upper graph) and *SLC8A1* mRNA (lower graph) were measured by PCR in the indicated cell lines. Each value is the mean ± SD of 3 independent cultures. (**B**) The panel illustrates the box plots generated from the median expression levels of *circSLC8A1* RNA and *SLC8A1* mRNA determined in *Lum*, *TNBC*, and *TNBC-mes* cell lines and it summarizes the results shown in (**A**). * Significantly lower in the Lum relative to the *TNBC-mes* group, as indicated (*p* = 0.050; Student’s T-test). (**C**) The panel illustrates the correlations between the mean expression of *circSLC8A1* RNA and *SLC8A1* mRNA in each cell line. The calculated R^2^ correlation value (Pearson method) and the correlation *p*-value are indicated. (**D**) Total RNA was extracted from the indicated cell lines and the levels of *circCCNB1* RNA (upper graph) and *CCNB1* mRNA (lower graph) were measured by PCR in the indicated cell lines. Each value is the mean ± SD of 3 independent cultures. (**E**) The panel illustrates the box plots generated from the median expression levels of *circCCNB1* RNA and *CCNB1* mRNA determined in *Lum*, *TNBC*, and *TNBC-mes* cell lines and it summarizes the results shown in (**D**). * Significantly higher in the *TNBC* relative to the *TNBC-mes* group, as indicated (*p* = 0.039; Student’s T-test). (**F**) The panel illustrates the correlations between the mean expression of *circCCNB1* RNA and *CCNB1* mRNA in each cell line. The calculated R^2^ correlation value (Pearson method) and the correlation *p*-value are indicated.

Within the group of 11 circRNAs showing upregulation in *mesHMLE* cells (Table 1), *circSLC8A1* and *circCCNB1* were found to be differentially expressed in the *Lum*, *TNBC*, and *TNBC-mes* groups (Figure 1). The levels of *circSLC8A1* were significantly higher in *TNBC-mes* than in *Lum* cell lines (Figure 1A,B).

In our panel of cell lines, the amounts of *circSLC8A1* and the linear *SLC8A1* transcript were quantitatively correlated (Figure 1C). As for *circCCNB1*, *TNBC* cells were found to contain larger amounts of this RNA than the *TNBC-mes* counterparts (Figure 1D,E). Once again, the levels of *circCCNB1* and linear *CCNB1* RNAs were correlated (Figure 1F). The expression of all other circRNAs, which are upregulated in *mesHMLE* cells (*circPVRL3*, *circSHPRH*, *circVEGFC*, *circBMPR2*, *circASXL1*, *circSMARCA5*, *circATXN2*, *circRTN4*, and *circSETD3*), did not show any difference in *Lum*, *TNBC*, and *TNBC-mes* cells (Figures S3–S6). In conclusion, *circSLC8A1* is the only upregulated circRNA whose expression profile in our breast cancer cell lines was found to be consistent with what is expected from the data obtained in *HMLE* and *mesHMLE* cells.

When we considered the four circRNAs characterized by downregulation in *mesHMLE* cells, *circGNB1*, *circFGD6*, and *circTNFRSF21* showed variable, albeit similar, levels of expression in *Lum*, *TNBC*, and *TNBC-mes* cell. lines (Figures S7 and S8). A superimposable expression pattern is evident in the case of the linear *GNB1*, *FGD6*, and *TNFRSF21* mRNAs.

**Figure 2:**
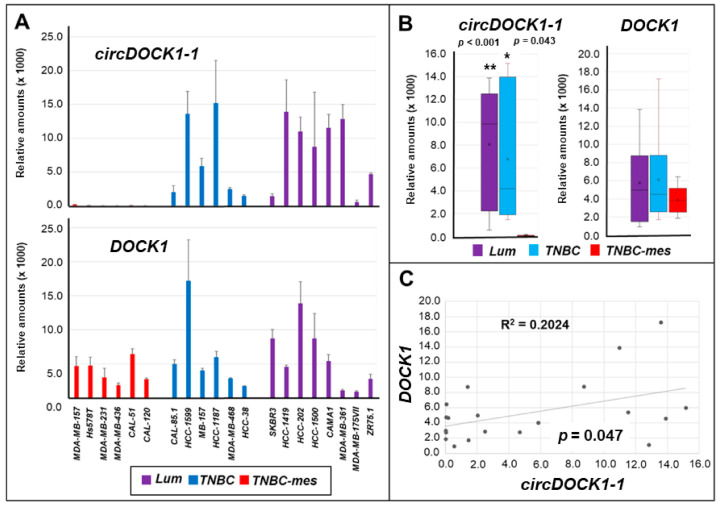
Expression of *circDOCK1-1* RNA and the corresponding linear *DOCK1* mRNA in breast cancer cell lines. Total RNA was extracted from the indicated cell lines and the levels of *circDOCK1-1* RNA (upper graph) and *DOCK1* mRNA (lower graph) were measured by PCR in the indicated cell lines (**A**). Each value is the mean ± SD of 3 independent cultures. (**B**) The panel illustrates the box plots generated from the median expression levels of *circDOCK1-1* RNA and *DOCK1* mRNA determined in *Lum*, *TNBC*, and *TNBC-mes* cell lines and it summarizes the results shown in (**A**). ** Significantly higher in the *Lum* relative to the *TNBC-mes* group, as indicated (*p* < 0.001; Student’s T-test). * Significantly higher in the *TNBC* relative to the *TNBC-mes* group (*p* = 0.043; Student’s *t*-test). (**C**) The panel illustrates the correlations between the mean expression of *circDOCK1-1* RNA and *DOCK1* mRNA in each cell line. The calculated R^2^ correlation value (Pearson method) and the correlation *p*-value are indicated.

As for *circDOCK1-1*, this is one of the 126 circular RNAs that can be produced from the *DOCK1* (*Dedicator of Cytokinesis 1*) gene (*hsa_circ_0020397*; Table S2). In fact, *DOCK1* is a very complex gene located on chromosome 10, consisting of 52 exons and coding for a series of variant proteins, which are guanine nucleotide exchange factors for the small Rho family G proteins [27,28,29]. The encoded proteins regulate the small GTPase Rac and influence various biological processes, including phagocytosis, cell migration, and proliferation. *circDOCK1-1* is generated by back-splicing of exon 27 to exon 2. We assumed the intervening exons 3–26 are spliced to also be included in *circDOCK1-1*, as illustrated in Figure S9, but because the circRNA is only identified by the sequence across the back-splice junction, this remains to be confirmed. *Lum* and *TNBC* cells were found to express significant amounts of *circDOCK1-1* (Figure 2A,B). In contrast, the *TNBC-mes* counterparts did not produce detectable levels of this circular-RNA, which is entirely consistent with *circDOCK1-1* downregulation in *mesHMLE* cells. The lack of *circDOCK1-1* expression in *TNBC-mes* cells is not due to the absence of the linear *DOCK1* transcript, whose quantities were substantially similar in the *TNBC-mes*, *Lum*, and *TNBC* cellular subtypes (Figure 2A,B). In this case, too, a significant (*p* = 0.047), though low, correlation between the expression levels of *circDOCK1-1* RNA and the corresponding linear transcript in the various cell lines was observed (Figure 2C).

### 3.2. Transcriptomic Perturbations Afforded by circDOCK1-1 Overexpression in TNBC-Mes Cells

**Figure 3:**
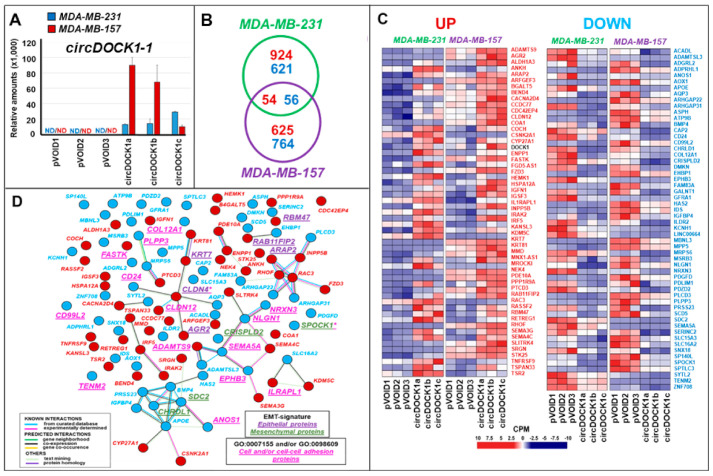
Transcriptomic effects of *circDOCK1-1* overexpression in *MDA-MB-231* and *MDA-MB-157* cells. Three independent *MDA-MB-231* and *MDA-MB-157* cell clones stably transfected with the *circDOCK1-1* expression plasmid (*circDOCK1a*; *circDOCK1b*; *circDOCK1c*) and the corresponding empty vector (*pVOID1*; *pVOID2*; *pVOID3*) were isolated and used for the experiments presented in the various panels of the figure. (**A**) The column graph shows the levels of the *circDOCK1-1* RNA determined by RT-PCR analysis of the total RNA extracted from the indicated cell populations using a specific Taqman assay. ND = not detectable. (**B**) The Venn diagram shows the number of genes significantly up- and downregulated by *circDOCK1-1* overexpression in *MDA-MB-231* and *MDA-MB-157* cells. Upregulated genes are marked in red and downregulated genes are marked in blue, as indicated. (**C**) The heat maps show the expression levels of the indicated genes measured in 3 independent clones of *MDA-MB-157* and *MDA-MB-231* cells transfected with a plasmid construct, allowing the expression of the *circDOCK1-1 RNA* and the corresponding void vector. The results were obtained using *RNA-seq* following isolation of total RNA and are expressed in CPM (Counts Per Million reads). The levels of *DOCK1* are shown and marked in black, as they represent an internal control of the experiment. In fact, the *DOCK1* reads are expected to increase in *circDOCK1-1* infected cells. (**D**) The panel shows the interactomic network of the proteins whose coding transcripts are upregulated (red circles) and downregulated (blue circles) in both *MDA-MB-231* and *MDA-MB-157* cells overexpressing *circDOCK1-1*. The proteins marked in violet are predominantly expressed in breast cancer tissues and cells characterized by an epithelial phenotype, while those marked in green are predominantly expressed in the mesenchymal counterparts, as indicated. The proteins involved in cell adhesion are marked in pink. The type of predicted interactions among the various proteins is also indicated by the coloured lines. CLDN* = *CLDN* is both an epithelial protein as well as a cell and cell/cell adhesion protein. SPOCK1* = *SPOCK1* is both a mesenchymal protein as well as a cell and cell/cell adhesion protein.

The results obtained suggest that *circDOCK1-1* exerts an onco-suppressive activity in triple-negative breast cancer cells. To directly support this idea, we transfected the *TNBC-mes MDA-MB-231* and *MDA-MB-157* cell lines with a *circDOCK1-1* construct and the corresponding empty vector (*pVOID*) as a negative control. Following selection in G418, we isolated three independent empty vector and *circDOCK1-1-*overexpressing cell clones (*pVOID1*; *pVOID2*; *pVOID3*; *circDOCK1a*; *circDOCK1b*; *circDOCK1c*) for each cell line. As expected, the *pVOID*-transfected *MDA-MB-231* and *MDA-MB-157* cells did not express detectable amounts of *circDOCK1-1* (Figure 3A). In contrast, the *MDA-MB-231* and *MDA-MB-157* cells transfected with the circRNA construct produced levels of *circDOCK1-1* that are similar to those detected in *Lum* and *TNBC* cell lines.

We subjected the *MDA-MB-231*- and *MDA-MB-157*-derived cells to *RNA-seq* analysis. In both *MDA-MB-231* and *MDA-MB-157* cells, where more than 24,000 RNAs were identified, *circDOCK1-1* overexpression caused limited perturbations on the gene expression profiles (Figure 3B). In *MDA-MB-231* cells, *circDOCK1-1* was found to upregulate 978 and downregulate 677 transcripts (Table S3 and Figure S10A). In *MDA-MB-157* cells, *circDOCK1-1* upregulates 679 and downregulates 820 mRNAs (Table S3 and Figure S10B). The *RNA-seq* results were validated in both *MDA-MB-231* and *MDA-MB-157* for two upregulated (*CYP27A1*; *SEMA3G*) and two downregulated (*IGFBP4*; *SDC2*) genes using PCR (Figure S11). *circDOCK1-1* overexpression upregulates 54 (including *DOCK1*) and downregulates 56 of these mRNAs in both *MDA-MB-231* and *MDA-MB-157* (Figure 3C and Table S3). Using the STRING database (https://string-db.org, accessed on 10 April 2021), we defined 10 “protein-interaction-networks”, five of which contain gene products involved in cell adhesion and cell–cell interactions (Figure 3D). This is consistent with the ssGSEA data that we obtained with the 110 genes regulated by *circDOCK1-1* in both *MDA-MB-231* and *MDA-MB-157* cells (Table S3) that show an enrichment of elements belonging to the GO:0007155 (Cell Adhesion) and GO:0098609 (Cell-cell Adhesion) *Gene Ontology* pathways.

As the ssGSEA results further indicate that the two top enriched pathways (GO:0010769 and GO:0022604) control cell morphogenesis and differentiation, we evaluated whether *circDOCK1-1* overexpression is associated with the induction of an epithelial phenotype. For this purpose, we investigated the epithelial/mesenchymal nature of the gene products commonly regulated by *circDOCK1-1* in *MDA-MB-231* and *MDA-MB-157* cells, using the EMT gene signatures determined in breast cancer [30]. *circDOCK1-1* caused a significant induction (Figure 3D) of various mRNAs coding for proteins annotated as epithelial (*AGR2*, *ARAP2*, *CLDN4*, *KRT7*, *RAB11FIP2,* and *RBM47*; see Supplementary Materials for the full name of the genes), whereas it downregulated different transcripts whose protein products are annotated as mesenchymal (*CHRDL1*, *SDC2*, *CRISPLD2*, and *SPOCK1*) (Figure 3D).

**Figure 4:**
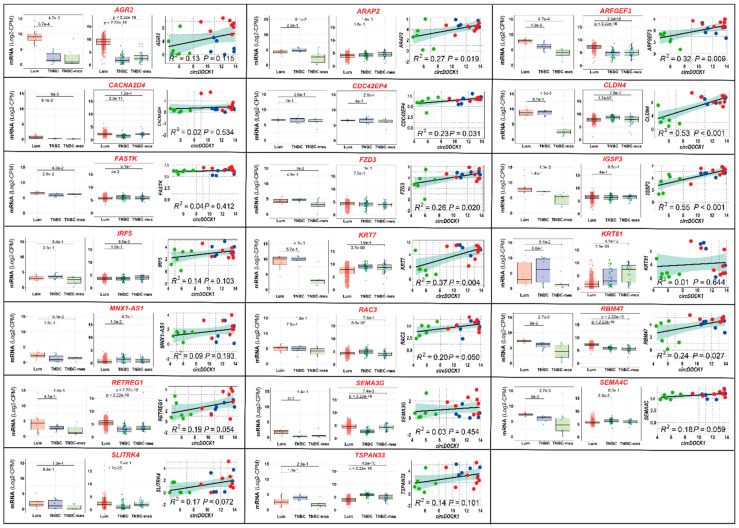
Expression profiles of some of the *circDOCK1-1* upregulated genes in breast cancer cell lines and tumour specimens. The indicated mRNAs and single non-coding RNA (*MNX-AS1*) are coded by some of the genes that are commonly upregulated in *MDA-MB-231* and *MDA-MB-157* cells overexpressing *circDOCK1-1*, according to our *RNA-seq* data. These RNAs are characterized by a trend towards higher expression levels in the *Lum* vs. *TNBC-mes* cell lines belonging to our panel of 18 cell lines (left diagrams of each panel; the *p*-values of the indicated comparisons are shown, Wilcoxon test). The expression profiles of the RNAs that can be determined from the *RNA-seq* data obtained from the breast cancer specimens available in the TCGA database are also illustrated (middle diagrams of each panel; the *p*-values of the indicated comparisons are shown, Wilcoxon test). The right diagrams of each panel show the quantitative correlations between the levels of the indicated mRNA and *circDOCK1-1* in each breast cancer cell line considered. *TNBC-mes*, *TNBC* and *Lum* cell lines are indicated by the green, blue, and red dots, respectively. The calculated R^2^ correlation value (Pearson method) and the correlation *p*-value are indicated.

To confirm the data, we evaluated the expression of the up-/downregulated transcripts in *TNBC-mes*/*TNBC*/*Lum* cell lines and established correlations between the levels of these mRNAs and *circDOCK1-1* in each cell line. As for the upregulated transcripts, the levels of 20 mRNAs, including *AGR2*, *ARAP2*, *CLDN4*, *KRT7*, and *RBM47*, tended to be lower in *TNBC-mes* than *Lum* cell lines (Figure 4, left box-plots). In 15 cases, this was accompanied by a significant direct correlation (R^2^ > 0.1) with *circDOCK1* expression in *TNBC-mes*, *TNBC*, and *Lum* cell lines (Figure 4, right diagrams). Moreover, the expression profiles of *AGR2*, *ARFGEF3*, *CACNA2D4*, *CDC42EP4*, *FZD3*, *RBM47*, *RETREG1*, and *SEMA3G* reflect the situation observed in primary mammary tumours (Figure 4, middle box-plots; TCGA *RNA-seq* data). Finally, the amounts of the 11 mRNAs tend to be larger in *TNBC-mes* than *Lum* cell lines (Figure S12), while 22 genes are characterized by similar levels of expression in *TNBC-mes* and *Lum* cell lines (Figure S13).

**Figure 5:**
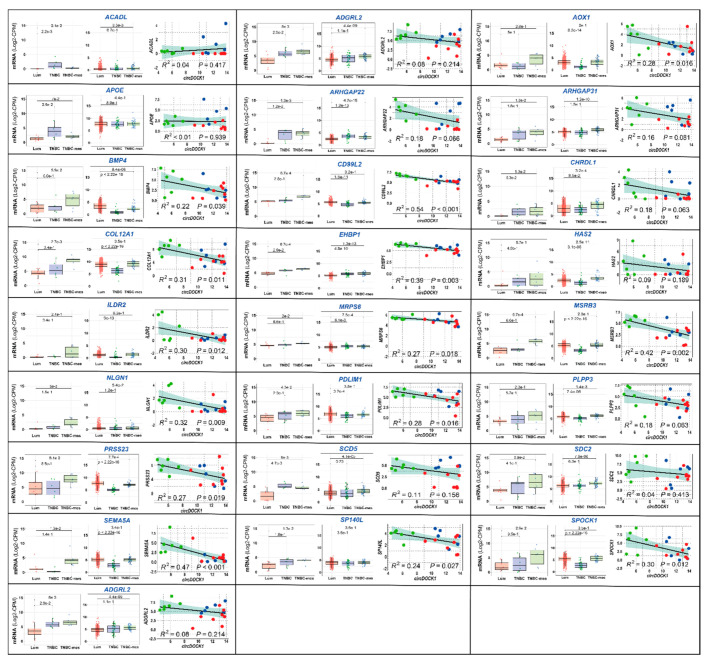
The expression profiles of some of the *circDOCK1-1* downregulated genes in breast cancer cell lines and tumour specimens. The indicated mRNAs are encoded by some of the genes that are commonly downregulated in MDA-MB-231 and *MDA-MB-157* cells overexpressing *circDOCK1-1*, according to our *RNA-seq* data. These mRNAs are characterized by a trend towards lower expression levels in the *Lum* vs. *TNBC-mes* cell lines belonging to our panel of 18 cell lines (left diagrams of each panel; the *p*-values of the indicated comparisons are shown, Wilcoxon test). The expression profiles of the mRNAs that can be determined from the *RNA-seq* data obtained from the breast cancer specimens available in the TCGA database are also illustrated (middle diagrams of each panel; the *p*-values of the indicated comparisons are shown, Wilcoxon test). The right diagrams of each panel show the quantitative correlations between the levels of the indicated mRNA and *circDOCK1-1* in each breast cancer cell line considered. *TNBC-mes*, *TNBC*, and *Lum* cell lines are indicated by the green, blue, and red dots, respectively. The calculated R^2^ correlation value (Pearson method) and the correlation *p*-value are indicated.

As for the transcripts that are downregulated by *circDOCK1-1* in *MDA-MB-231* and *MDA-MB-157* cells, the levels of 25 mRNAs tended to be higher in *TNBC-mes* than *Lum* cell lines (Figure 5, left boxplots). For the majority of these transcripts, the situation reflects what is observed in primary breast cancer specimens (Figure 5, middle boxplots). Only four of these mRNAs (*ADGRL2*, *APOE*, *HAS2,* and *SDC2*) are devoid of a significant inverse correlation with *circDOCK1* (Figure 5, right diagrams; R^2^ < 0.1). In contrast, the levels of six mRNAs tend to be lower in *TNBC-mes* than *Lum* cells (Figure S14), while 24 transcripts show similar levels in *TNBC-mes* and *Lum* cells (Figure S15).

Overall, the results obtained support the idea that approximately one-third of the genes that are upregulated by *circDOCK1-1* overexpression show higher levels in *Lum* and/or *TNBC* cells relative to the *TNBC-mes* counterparts, while a similar proportion of *circDOCK1-1* downregulated genes are characterized by an opposite expression profile. The data are consistent with a potential involvement of this set of genes in the process of epithelial differentiation triggered by *circDOCK1-1*.

### 3.3. Effects of circDOCK1-1 Knockdown on the Gene Expression Profiles of Luminal CAMA-1 Cells

**Figure 6:**
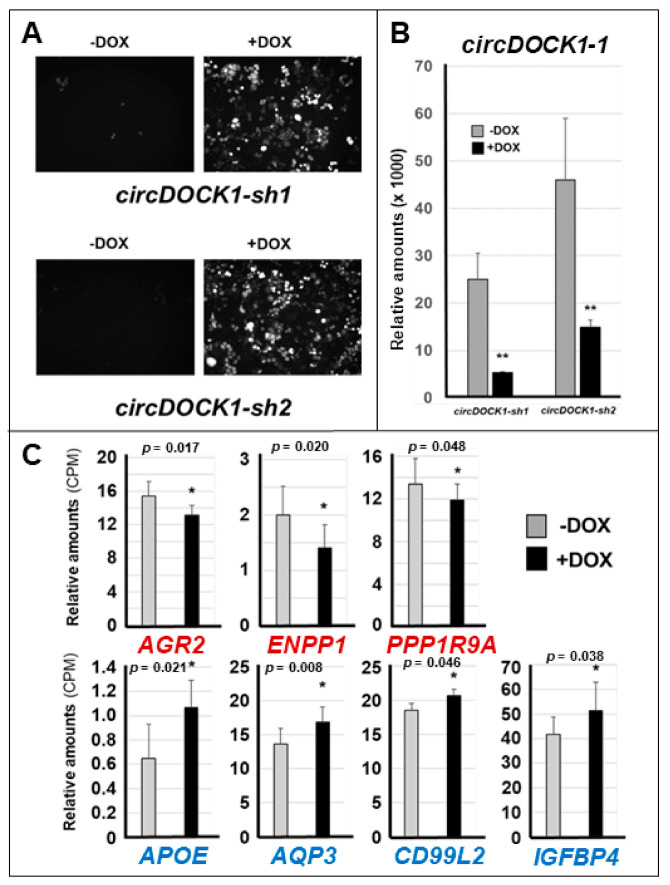
Effects of *circDOCK1-1* silencing in *CAMA-1* cells on the expression of genes whose expression is modulated by *circDOCK1-1* overexpression. Luminal *CAMA-1* cells were infected with a retroviral construct, which allows doxycyclin (DOX)-dependent silencing of *circDOCK1-1*. Following infection, two independent cell populations (*circDOCK1-sh1* and *circ-DOCK1-sh2*) were isolated. (**A**) The figures show that the two cell populations express significant levels of the *circDOCK1-1* targeting constructs only following exposure to DOX, as indicated by the positivity of cells to the Red Fluorescent Protein. (**B**) The column diagram shows the level of *circDOCK1-1* expression as determined by RT-PCR analysis with a specific Taqman assay. ** Significantly lower than the corresponding vehicle-treated (−DOX) sample (*p* < 0.01 following Student’s *t*-test). No significant difference was observed between the −DOX *circDOCK1-sh1* and *circ-DOCK1-sh2* groups. (**C**) The column graphs shows: (1) the levels of the indicated mRNAs that are significantly upregulated by *circDOCK1-1* overexpression in both *MDA-MB-231* and *MDA-MB-157* cells and are marked in red (*AGR2*, *MPP1*, and *PPP1R9A*); (2) the levels of the indicated mRNAs that are significantly downregulated by *circDOCK1-1* overexpression in both *MDA-MB-231* and *MDA-MB-157* cells and are marked in blue (*APOE*, *AQP3*, *CD99L2*, and *IGFBP4*). The results were obtained using *RNA-seq* following isolation of total RNA from triplicate cultures of the two cell populations exposed to vehicle or DOX and are expressed in CPM (counts per million reads). * Significantly different. The *p*-values of the −DOX vs. +DOX comparisons (Student’s *t*-test; N = 6) are shown.

To obtain insights into the influence played by the luminal/mesenchymal phenotypes on the expression of *circDOCK1-1-*regulated genes, we silenced *circDOCK1-1* in *Lum CAMA-1* cells, which produce large amounts of endogenous *circDOCK1-1* (Figure 2). *CAMA-1* cells were infected with retroviral constructs containing two independent doxycycline (DOX)-inducible shRNAs targeting *circDOCK1-1*. Following infection, we isolated two puromycin-resistant *CAMA-1* cell populations (*circDOCK-sh1* and *circDOCK-sh2*) expressing each construct (Figure 6A). In these cellular populations, the production of the two shRNAs was monitored by the RFP (Red-Fluorescent-Protein) marker, whose expression is also DOX inducible (Figure 6A). As expected, DOX caused a significant downregulation of the *circDOCK1-1* RNA in *circDOCK-sh1* and *circDOCK-sh2* cells (Figure 6B).

The two *circDOCK-sh1* and *circDOCK-sh2* cell lines were used to conduct *RNA-seq* studies in the presence/absence of DOX. The data obtained demonstrate that DOX upregulates 527 and downregulates 490 out of the 21,965 RNAs, which can be identified in the two cell lines (unadjusted *p*-value ≤ 0.05; Table S4). Among the 110 mRNAs whose expression is modulated by *circDOCK1-1* overexpression in *MDA-MB-231* or *MDA-MB-157* cells, only *AGR2*, *ENPP1*, *PPP1R9A*, *APOE*, *AQP3*, *CD99L2*, and *IGFBP4* show a reversed regulation in *circDOCK1-1-*silenced *CAMA1* cells (Figure 6C). Indeed, *AGR2*, *ENPP1*, and *PPP1R9A* are up- and downregulated, respectively, by *circDOCK1-1* overexpression and *circDOCK1-1* silencing, while the opposite is true in the case of *APOE*, *AQP3*, *CD99L2*, and *IGFBP4*. *AGR2*, *ENPP1*, *PPP1R9A APOE*, *AQP3*, and *IGFBP4* are part of various common gene networks involved in cell differentiation and development (Table S3). This is consistent with the involvement of the corresponding gene products in the processes of epithelial differentiation activated by *circDOCK1* in mesenchymal *MDA-MB-157* and *MDA-MB-231* cells. To define potential off-target effects, we measured the levels of *DOCK1* mRNA in *CAMA1* cells exposed to vehicle (−DOX) and DOX (+DOX). We did not observe any significant difference in the expression levels of the *DOCK1* transcript (−DOX = 128.8 ± 5.3 CPM; +DOX = 117.2 ± 16.2 CPM; *p*-value (+DOX vs. −DOX = 0.146). These data support the idea that *circDOCK1-1* knockdown does not lead to significant off-target effects.

### 3.4. Involvement of circDOCK1-1 in the Growth, Differentiation, and Motility of Breast Cancer Cells

**Figure 7:**
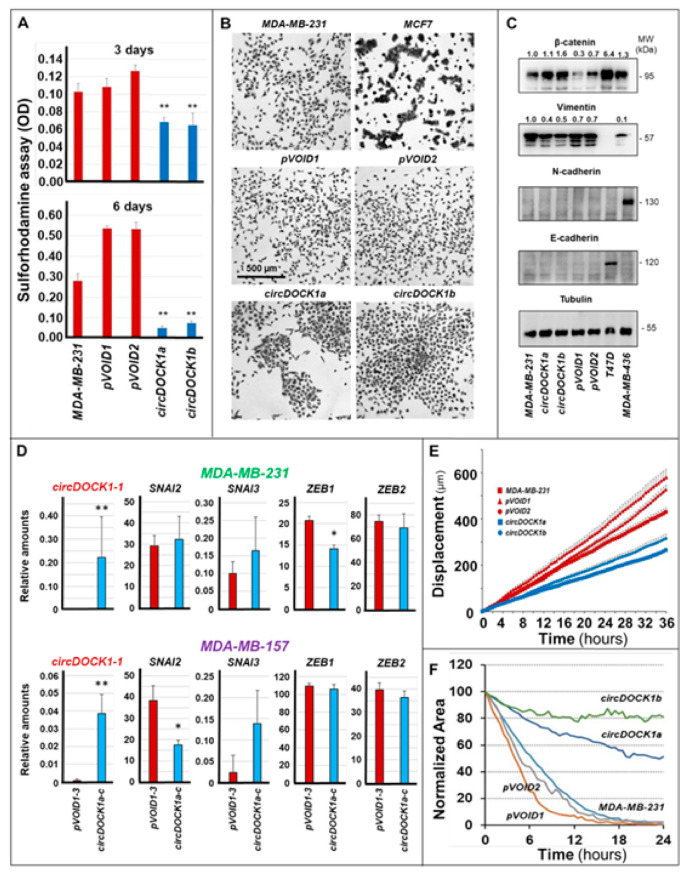
Effects of *circDOCK1-1* overexpression on cell proliferation, morphology, EMT, random motility, and wound healing. Parental *MDA-MB-231* as well as three derived cell populations stably transfected with a plasmid allowing the expression of *circDOCK1-1* (*circDOCK1a*; *circDOCK1b*; *circDOCK1c*) and the corresponding void vector (*pVOID1*; *pVOID2*; *pVOID3*) were used for the experiments described in the various panels. (**A**) The proliferation of parental *MDA-MB-231*, *pVOID1*, *pVOID2*, *circDOCK1a*, and *circDOCK1b* cells was evaluated with the sulforhodamine assay. ** Significantly lower than parental *MDA-MB-231*, *pVOID1*, and *pVOID2* (*p* < 0.001 Student’s *t*-test). (**B**) The images show the morphology of logarithmically growing parental *MDA-MB-231*, *pVOID*1, *pVOID2*, *circDOCK1a*, and *circDOCK1b* cells grown in optimal conditions on plastic dishes. Luminal *MCF7* cells grown in the presence of oestradiol were used as a positive control for the luminal phenotype. (**C**) Total cellular extracts obtained from parental *MDA-MB-231*, *pVOID1*, *pVOID2*, *circDOCK1a*, and *circDOCK1b* cells grown under basal conditions were used to perform Western blot experiments with specific antibodies recognizing the indicated proteins. Protein extracts of the luminal T47D and the mesenchymal *MDA-MB-436* cell lines were used as external controls for the experiments. The same amount of protein was loaded in each lane as indicated by the levels of tubulin shown. The numbers above each lane of the β-catenin and vimentin Western blots indicate the relative levels of the two proteins after densitometric analysis of the bands taking the values determined in parental *MDA-MB-231* cells 1.0. All the values were normalized for the corresponding amounts of tubulin. (**D**) The diagrams illustrate the expression levels of the indicated mRNAs coding for proteins playing a positive action in the process of epithelial-to-mesenchymal transition (EMT) determined from the RNA-seq data obtained in the indicated *pVOID1*, *pVOID2*, *pVOID3*, *circDOCK1a*, *circDOCK1b*, and *circDOCK1c* populations. Each value is the Mean ± SD of the three indicated cell populations. ** Significantly different from the corresponding *pVOID* value (*circDOCK1-1* in *MDA-MB-231* cells, *p* < 0.001; *circDOCK1-1* in *MDA-MB-157* cells, *p* < 0.001; Student’s *t*-test). * Significantly different from the corresponding *pVOID* value (*ZEB1* in *MDA-MB-231* cells, *p* = 0.028; *SNAI2* in *MDA-MB-157* cells, *p* = 0.012; Student’s *t*-test). (**E**) The diagrams illustrate the random motility curves determined in parental *MDA-MB-231*, *pVOID1*, *pVOID2*, *circDOCK1a*, and *circDOCK1b* cells by time-lapse microscopy at the indicated time points. The motility curves of *circDOCK1a* and *circDOCK1b* cells are significantly lower than the corresponding curves generated from the data obtained with parental *MDA-MB-231*, *pVOID1*, and *pVOID2* cells (*p* < 0.001 following two-way ANOVA Bonferroni post-test). (**F**) The diagrams show the wound-healing curves determined in parental *MDA-MB-231*, *pVOID1*, *pVOID2*, *circDOCK1a*, and *circDOCK1b* cells by time-lapse microscopy at the indicated time points. The values indicate the void area contained between the margins of the healing. The curves obtained with *circDOCK1a* and *circDOCK1b* cells indicate that the closure of the wound was slower than what was observed with parental *MDA-MB-231, pVOID1* and *pVOID2* cells (*p* < 0.001 following two-way ANOVA Bonferroni post-test).

The data obtained in breast cancer cells indicate that *circDOCK1-1* is involved in the generation/maintenance of the epithelial phenotype, which is characterized by a decrease in the proliferation rate and motility as well as an increase in cell–cell interactions relative to the mesenchymal counterpart. Thus, we evaluated the effects of *circDOCK1-1* in our genetically engineered *MDA-MB-231* cells. In basal conditions, control *pVOID1*/*pVOID2* and parental *MDA-MB-231* cells show similar growth rates (Figure 7A). In the same situation, the basal growth of *circDOCK1a* and *circDOCK1b* cells overexpressing *circDOCK1-1* is substantially reduced. In addition, *pVOID1*/*pVOID2* and parental *MDA-MB-231* cells grow in a scattered fashion and show the characteristic elongated structure of mesenchymal cells (Figure 7B). In contrast, *circDOCK1a* and *circDOCK1b* cells tend to form aggregates due to increased cell–cell interactions and show an epithelial-like morphology, which is typical of breast cancer *Lum* cells, as exemplified by the *MCF7* cell line.

The results described above support the concept that *circDOCK1-1* overexpression in *TNBC-mes* cells activates a process of epithelial differentiation. Consistent with this idea, β-catenin, an epithelial protein often upregulated in luminal breast cancer [31,32], was significantly induced by *circDOCK1-1* overexpression in *MDA-MB-231* cells (Figure 7C). Although *MDA-MB-231* cells do not express detectable amounts of the mesenchymal marker, N-cadherin, it is interesting to notice that *circDOCK1a*/*circDOCK1b* cells showed lower levels of vimentin, another mesenchymal marker protein [33], than *pVOID1*/*pVOID2* cells (Figure 7C). This is consistent with the idea that *circDOCK1-1* is indeed inducing an epithelial phenotype. Nevertheless, the process of epithelial differentiation ignited by the circular RNA in the *TNBC-mes* cell line is likely to be incomplete, as E-cadherin, a late epithelial markers [34], was undetectable in either *pVOID1*/*pVOID2* or *circDOCK1a*/*circDOCK1b* cells (Figure 7C). Moreover, endogenous *circDOCK1-1* is unlikely to induce an epithelial phenotype via downregulation of any of the four transcription factors that activate EMT, i.e., *SNAI2*, *SNAI3*, *ZEB1*, and *ZEB2* [35,36,37]. Indeed, the *RNA-seq* data indicate that *circDOCK1-1* does not alter the expression of *SNAI3* and *ZEB2* in either the *MDA-MB-231* or the *MDA-MB-157* cellular context, while it downregulates *SNAI2* only in *MDA-MB231* cells and *ZEB1* only in *MDA-MB-157* cells (Figure 7D).

Epithelial differentiation and increased cell–cell interactions are generally accompanied by effects on random and directional motility, which are primary determinants of the invasive/metastatic behaviour of mesenchymal breast cancer cells. Consistent with the observed effects of *circDOCK1-1* on differentiation/adhesion, *circDOCK1a*/*circDOCK1b* cells showed a significant reduction in random motility relative to the parental *MDA-MB-231* cells and *pVOID1*/*pVOID2* controls (Figure 7E), as indicated by time-lapse assays. A similar decrease in the directional motility of *circDOCK1a* and *circDOCK1b* cells was observed when the various *MDA-MB-231*-derived cell populations were subjected to wound-healing assays. Indeed, the wound was entirely closed by parental and *pVOID1*/*pVOID2* cells within 24 h, while less than 50% and 20% of the wound area was filled by *circDOCK1a*/*circDOCK1b* cells (Figure 7F). The results obtained on random and directional motility were validated by similar studies performed with an independent *MDA-MB-231* clone transfected with *circDOCK1-1* (*circDOCK1c*) and empty (*pVOID3*) vectors (Figure S16A,B).

## 4. Discussion

A better definition of the role played by specific circular RNAs in the growth and progression of solid tumours with particular reference to the different types of breast cancer is of great relevance. In fact, this type of information is likely to provide new clues for the design of innovative strategies in the context of the stratified/personalized treatment of mammary tumours, which are a highly heterogeneous collection of different diseases.

In the present study, we identified one of the 126 circular RNAs (Table S2; http://www.circbase.org/, accessed on 12 May 2021) that originate from the *DOCK1* gene (*circDOCK1-1*; *hsa_circ_0020397*) as a positive regulator of the epithelial differentiation of mammary tumour cells. Indeed, the data obtained in a large panel of cell lines, recapitulating the heterogeneity of breast cancer, indicate that *circDOCK1-1* is undetectable in cultures of *TNBC* cells with a mesenchymal phenotype (*TNBC-mes*), while it is highly expressed in *Lum* and *TNBC* cells characterized by an epithelial phenotype.

Forced expression of *circDOCK1-1* in two representative *TNBC-mes* cell lines (*MDA-MB-231*; *MDA-MB-157*) caused the upregulation of genes encoding epithelial proteins and the downregulation of genes encoding mesenchymal proteins. Our *RNA-seq* data indicate that *circDOCK1-1* induces and maintains certain traits of the epithelial phenotype in breast cancer cells via the direct or indirect regulation of approximately 100 target genes.

A significant proportion of the genes up- and downregulated by *circDOCK1-1* in *MDA-MB-231* and *MDA-MB-157* cells code for proteins that control cell-to-cell contacts and cell adhesion, two parameters that are generally increased in luminal/epithelial relative to mesenchymal breast cancer cells. Among the genes upregulated by *circDOCK1* in the two *TNBC-mes* cells and belonging to the protein-interactome networks identified (Figure 3D), *ARAP2* and *IRF5* are of particular interest. *ARAP2* codes for an epithelial protein involved in the formation of focal adhesions [38]. *ARAP2* upregulation suggests that *circDOCK1-1* stimulates the generation of focal adhesions, which connect the actin cytoskeleton with the extracellular matrix and play a critical role in cell survival, proliferation, and movement. *IRF5* is a transcription factor and its loss increases the motility and contributes to the activation of the metastatic behaviour of breast cancer cells [39,40]. Among the downregulated genes, *EPHB3* (*EPH-receptor-B3*) is of particular relevance. EPHB3 is an ephrin-binding membrane receptor, which facilitates cell adhesion [41], enhancing the migration and promoting the metastatic behaviour of lung cancer cells [42]. In addition, high EPHB3 levels are associated with poor survival in breast cancer patients, which supports its oncogenic and pro-metastatic properties [43]. To restrict the number of genes that may be causally involved in *circDOCK1-1*-induced epithelial differentiation, we performed silencing experiments in a *Lum* cell-line (*CAMA-1*) characterized by high levels of *circDOCK1-1* expression. The *RNA-seq* studies performed in this model resulted in the identification of seven genes: *AGR2*, *ENPP1*, *PPP1R9A*, *APOE*, *AQP3*, *CD99L2*, and *IGFBP4*, whose expression pattern is opposite to that observed upon *circDOCK1-1* overexpression. *circDOCK1-1* knockdown increases the levels of the *AGR2*, *ENPP1*, and *PPP1R9A* transcripts, while it decreases the amounts of the *APOE*, *AQP3*, *CD99L2*, and *IGFBP4* mRNAs. *AGR2*, *APOE*, *AQP3*, and *CD99L2* may be of particular interest for the epithelial differentiating action exerted by *circDOCK1-1* in *TNBC-mes* breast cancer cells. *AGR2* is a protein disulfide isomerase [44] and it is expressed almost exclusively in luminal breast tumours [45], where it is positively regulated by the oestrogen receptor. In the mammary gland, *AGR2* is believed to promote lobulo-alveolar development, which is consistent with the role exerted in the intestine, where the protein is implicated in maintaining the epithelial barrier [46]. *APOE* controls the differentiation and morphogenesis of neuronal cells [47], while *AQP3* may have a role in kidney development [48]. *CD99L2* is encoded by one of the six mesenchymal genes, which are part of an EMT signature in ovarian carcinoma [49]. In mesenchymal-like ovarian cancer cells, silencing of *CD99L2* causes colony compaction, indicating that the protein plays a negative role in cell-to-cell adhesion.

As for the potential mechanisms underlying the regulatory action of *circDOCK1-1* on the expression of a large number of target genes identified in our study, protein and microRNA sponging effects may be considered. Indeed, one of the hypothesized mechanisms underlying the gene regulatory action of circular RNAs is related to the ability of these molecules to act as sponges for specific proteins and microRNAs [50,51]. To obtain initial insights into the problem, we focused our attention on microRNAs that may exert a regulatory action on the *AGR2*, *ENPP1*, and *PPP1R9A* genes, which are upregulated by forced expression and silencing of *circDOCK1-1*. In fact, our data are consistent with the idea that *circDOCK1-1* may be a direct regulator of these genes in breast cancer cells. To predict microRNAs that may bind each of the three mRNAs mentioned above and *circDOCK1-1* contemporaneously, we used an in silico approach (http://mirdb.org/mirdb/custom.html, accessed on 10 September 2021). The results of this analysis (Table S5) indicate that the *AGR2*, *ENPP1*, and *PPP1R9A* mRNAs have the potential to bind four, eight, and four microRNAs, which can interact with the *circDOCK1-1* RNA as well. Noticeably, the transcriptomic results available in the CCLE and TCGA databases support the idea that the only microRNAs for which there is evidence of expression in breast cancer cell lines or tissues are *hsa-mir-539-3p* and *hsa-miR-545-3p*. Thus, *circDOCK1-1* may increase the expression of *AGR2*, *ENPP1*, and *PPP1R9A* transcripts via a sponging effect exerted on *hsa-mir-539-3p*, *hsa-miR-545-3p*, and/or some of the other microRNAs identified in Table S5 Clearly, further functional studies are needed to confirm the hypothesized molecular mechanism of action and the potential involvement of microRNAs in the process of epithelial differentiation activated by *circDOCK1-1* in *TNBC-mes* breast cancer cells.

Taken together, the results obtained in this study suggest that *circDOCK1-1* is endowed with antioncogenic properties in breast cancer, as it seems to favour epithelial differentiation and reduce the proliferation as well as the motility/invasiveness of tumour cells characterized by a mesenchymal phenotype. To the best of our knowledge, no data are available on the expression levels or functional activity of any of the 126 *circDOCK1* RNAs, including *circDOCK1-1*, in triple-negative breast cancer. However, a certain amount of relevant data on the functional role played by *circDOCK1* RNAs in other types of tumours is present in the scientific literature. As an example, Zhang et al. recently reported that *circDOCK1-1* expression is high in colorectal cancer cells and forced expression of the circular RNA increased the viability and invasive properties of cells derived from this tumour, suggesting a pro-oncogenic action [52]. This may support the idea that the role played by *circDOCK1-1* in regulating the homeostasis of cancer cells is tumour type specific. To further complicate the issue, it should be noticed that another member of the *DOCK1* family of circular RNAs, i.e., *hsa_circ_0020394* (see Table S2), has been shown to promote the progression of bladder cancer via the has-miR-132-3p/*SOX5* signalling pathway [53]. All this is likely to spur interest in the conduction of studies aimed at elucidating the physio-pathological function of *circDOCK1-1* and other members of this circular-RNA family in breast cancer and other tumour types.

## 5. Conclusions

The results of the study are of basic interest, as they provide novel insights into the role played by specific circRNAs in the generation and progression of breast cancer. In fact, our data identified *circDOCK1-1* as an important regulator of the epithelial differentiation of mammary tumour cells. In addition, the high-throughput transcriptomic studies performed provide information on the number and type of genes that are regulated by *circDOCK1-1*, casting new light on the gene networks involved in the processes underlying the development of mammary tumours. Our results further indicate that *circDOCK1-1* controls cell–cell adhesion and consequently random as well as directional motility. Besides their interest from a basic point of view, the data generated may be of translational relevance, as they suggest that strategies aimed at increasing the amounts or at stimulating the activity of *circDOCK1-1* may be of significance for the treatment of a subset of triple-negative breast cancer patients.

## Figures and Tables

**Table 1:** Differential expression of specific circRNA in mammary gland epithelial cells following EMT. The expression of the indicated circRNA was determined after *RNA-seq* analysis of total RNA extracted from the human mammary gland epithelial cell line, control *HMLE* cells, and *HMLE* cells exposed to TGFβ (*HMLE-mes*) to induce EMT (epithelial-to-mesenchymal transition). The results are expressed as the mean counts determined in 2 independent samples as shown. The identification codes and the structural characteristics of the indicated circRNAs were obtained from the circBASE website (http://www.circbase.org/, accessed on 12 May 2021).

Circular RNA	Circ ID	Location	Exons (no)	Size (nt)	*HMLE* (Mean-Counts)	*HMLE-mes* (Mean-Counts)	*HMLE-mes*/*HMLE* (Fold Change)
*circPVRL3*	*hsa_circ_0066776*	chr3: 110830876-110845182	2-5 (4)	909	ND (0;0)	51 (68;34)	ND
*circSHPRH*	*hsa_circ_0001649*	chr6: 146209155-146216113	2-5 (4)	440	ND (0;0)	18 (21;16)	ND
*circVEGFC*	*hsa_circ_0004496*	chr4: 177632652-177650900	4-6 (3)	557	5 (11;0)	65 (91;39)	13.0
*circBMPR2*	*hsa_circ_0003218*	chr2: 203329531-203332412	2-3 (2)	342	21 (37;5)	102 (133;72)	4.9
*circCCNB1*	*hsa_circ_0001495*	chr5: 68470703-68471364	6-7 (2)	378	20 (24;16)	90 (114;66)	4.5
*circSLC8A1*	*hsa_circ_0000994*	chr2: 40655612-40657444	9 (1)	1832	67 (52;82)	271 (206;337)	4.0
*circASXL1*	*hsa_circ_0001136*	chr20: 30954186-30956926	2-3 (2)	195	101 (134;69)	375 (643;107)	3.7
*circSMARCA5*	*hsa_circ_0001445*	chr4: 144464661-144465125	15-16 (2)	269	47 (72;22)	174 (154;195)	3.7
*circATXN2*	*hsa_circ_0002457*	chr12:111990083-111993723	21-24 (4)	320	21 (35;8)	68 (85;51)	3.2
*circRTN4*	*hsa_circ_0001006*	chr2: 55209650-55214834	5-6 (2)	347	48 (47;49)	152 (152;152)	3.2
*circSETD3*	*hsa_circ_0000567*	chr14: 99924615-99932150	8-12 (5)	683	90 (115;65)	256 (209;304)	2.8
*circDOCK1-1*	*hsa_circ_0020397*	chr10:128768965-128926028	2-27 (26)	2738	172 (211;134)	7 (12;3)	0.04
*circGNB1*	*hsa_circ_0008702*	chr1: 1747194-1770677	8-11 (4)	298	38 (36;40)	13 (19;8)	0.3
*circFGD6*	*hsa_circ_0141065*	chr12: 95602618-95605043	20 (1)	2425	66 (57;74)	27 (29;25)	0.4
*circTNFRSF21*	*hsa_circ_0001610*	chr6: 47251673-47254331	4-5 (2)	1147	58 (65;52)	27 (22; 32)	0.5

## Data Availability

All the data presented are available in the manuscript and/or Supplementary Files provided. As indicated in the Materials and Methods section, the *RNA-seq* data have been deposited in the publicly available EMBL-EBI Arrayexpress database (Accession No: E-MTAB-10819).

## Supplementary Materials

The following are available online at https://www.mdpi.com/article/10.3390/cancers13215325/s1, Supplementary Methods, Figure S1: Principal Component Analysis of the RNA-seq data obtained in a panel of selected breast cancer cell-lines, Figure S2: Morphological features of representative cell-lines belonging to the Lum, TNBC and TNBC-mes groups, Figure S3: Clustering of the breast cancer cell-lines according to single-sample GSEA, Figure S4: Breast cancer cell-lines expression profiles of the circRNAs up-regulated in HMLE-mes cells, Figure S5: Expression of circPVRL2, circSHPRH, circVEGFC circRNAs and PVRL2, SHPRH, VEGFC linear mRNAs in our panel of breast cancer cell-lines, Figure S6: Expression of circBMPR2, circASXL1, circSMARCA5 circRNAs and BMPR2, ASXL1, SMARCA5 mRNAs in our panel of breast cancer cell-lines, Figure S7: Expression of circATXN2, circRTN4, circSETD3 circRNAs and ATXN2, RTN4, SETD3 mRNAs in breast cancer cell-lines, Figure S8: Breast cancer cell-lines expression profiles of the circRNAs down-regulated in HMLE-mes cells, Figure S9: Expression of circGNB1, circFGD6, circTNFRSF21 circRNAs and GNB1, FGD6, TNFRSF21 mRNAs in breast cancer cell-lines, Figure S10: Effects of circDOCK1 over-expression on the gene expression profiles of the MDA-MB231 and MDA-MB-157 cell-lines, Figure S11: Validation of the RNA-seq results on two up-regulated and two down-regulated genes by PCR analysis, Figure S12: Expression profiles of some of the genes up-regulated by circDOCK1 in breast cancer cell-lines and tumor specimens, Figure S13: Expression profiles of some of the genes up-regulated by circDOCK1 in breast cancer cell-lines and tumor specimens, Figure S14: Expression profiles of some of the genes down-regulated by circDOCK1in breast cancer cell-lines and tumor specimens, Figure S15: Expression profiles of some of the genes down-regulated by circDOCK1 in breast cancer cell-lines and tumor specimens, Figure S16: Effects of circDOCK1 over-expression on random motility and wound-healing, Table S1: RNA-seq analysis of the breast cancer cell-lines grown under basal conditions, Table S2: RNA-seq analysis of the perturbations afforded by forced expression of circDOCK1 in the MDA-MB-231 and MDA-MB-157 breast cancer cell-lines, Table S3: RNA-seq analysis of the perturbations afforded by circDOCK1 silencing in the CAMA1 breast cancer cell-line, Table S4: List of microRNAs predicted to interact with circDOCK1, Table S5: microRNAs predicted to bind the circDOCK1-1 RNA, the AGR2, ENPP1 and the PPP1R9A mRNAs.
