# Supplementary material for: A DOCK1 Gene-Derived Circular RNA Is Highly Expressed in Luminal Mammary Tumours and Is Involved in the Epithelial Differentiation, Growth, and Motility of Breast Cancer Cells
Source: Cancers (Basel). 2021 Oct 23;13(21):5325. doi: 10.3390/cancers13215325 (PMC8582367; doi:10.3390/cancers13215325)
Supplement: Supplementary file 1 [file cancers-13-05325-s001.zip › SUPPLEMENTARY_crc_rev2.pdf]

SUPPLEMENTARY INFORMATION

Title: A *DOCK1* gene derived circular RNA is highly expressed in luminal mammary tumors and it is involved in the epithelial differentiation, growth and motility of breast cancer cells

Mami Kurosaki, Mineko Terao, Dawei Liu, Adriana Zanetti, Luca Guarrera, Marco Bolis, Maurizio Gianni', Gabriela Paroni, Gregory J Goodall and Enrico Garattini

Supplementary Methods	page	2-9
Legends to Supplementary Tables S1-S5	page	10-13
Supplementary Figure S1	page	14
Supplementary Figure S2	page	15
Supplementary Figure S3	page	16
Supplementary Figure S4	page	17
Supplementary Figure S5	page	18
Supplementary Figure S6	page	19
Supplementary Figure S7	page	20
Supplementary Figure S8	page	21
Supplementary Figure S9	page	22
Supplementary Figure S10	page	23
Supplementary Figure S11	page	24
Supplementary Figure S12	page	25
Supplementary Figure S13	page	26
Supplementary Figure S14	page	27
Supplementary Figure S15	page	28
Supplementary Figure S16	page	29
Original Western blots Fig. 7C	page	30

SUPPLEMENTARY METHODS

Cell lines: The table below summarizes the characteristics and the source of the cell lines used throughout the study:

**Cell name**	**Gender**	**Phenotype**	**Source**
HCC-1599	F	Basal (triple-negative)	ATCC
MB-157	F	Basal (triple-negative)	ATCC
MDA-MB-468	F	Basal (triple-negative)	ATCC
HCC-1187	F	Basal (triple-negative)	ATCC
HCC-38	F	Basal (triple-negative)	ATCC
CAL85.1	F	Basal (triple-negative)	DSMZ
SKBR3	F	Luminal (HER2+)	ATCC
MDA-MB-175VII	F	Luminal (ER+)	ATCC
HCC-202	F	Luminal (HER2+)	ATCC
ZR75.1	F	Luminal (ER+)	ATCC
MDA-MB-361	F	Luminal(ER+/HER2+)	ATCC
HCC-1419	F	Luminal (HER2+)	ATCC
CAMA1	F	Luminal (ER+)	ATCC
HCC-1500	F	Luminal (ER+)	ATCC
MDA-MB-157	F	Basal (triple-negative)	ATCC
MDA-MB-436	F	Basal (triple-negative)	ATCC
CAL-120	F	Basal (triple-negative)	DSMZ
CAL-51	F	Basal (triple-negative)	DSMZ
MDA-MB-231	F	Basal (triple-negative)	ATCC
Hs578T	F	Basal (triple-negative)	ATCC
HMLE	F	Normal mammary gland	ATCC

ATCC = American Type Culture Collection; DSMZ = Deutsche Sammlung von Mikroorganismen und Zellkulturen; SIGMA = Sigma-Aldrich. The cell-lines marked in yellow are the 16 cell-lines

used to perform the RNA-seq experiments following treatment with ATRA. During the course of the entire study, all the cell lines were authenticated by constantly checking the morphology and the growth doubling time. In addition, all the cell lines were mycoplasma free, as indicated by periodic PCR assays performed on the cell conditioned medium, using the following nucleotide mycoplasma recognizing primers (forward: 5'TGCACCATCTGTCACTCTGTTAACCTC3'; reverse: 5'ACTCCTACGGGAGGCAGCAGTA3').

PCR studies: The primers used for the amplification of the circRNAs and corresponding linear mRNAs are listed in the Table below (F = forward amplimer; R = reverse amplimer). circRNAs were amplified from total RNA preparation after generation of the corresponding cDNA.

gene	primer name	circ specific primer sequence	primer name	mRNA primer sequence
SLC8A1	SLC8A1_circ1_F	TGATGAAATTGTTAGGTTGTGACAG	SLC8A1_mRNA_F	CATCAGTGCCAGACACATTTGC
	SLC8A1_circ1_R	TGGTCCACATGGGAAAATAAGAGA	SLC8A1_mRNA_R	CACACCGATTCCCAGGAAGA
SMARCA5	SMARCA_circF1	CTCCAAGATGGGCGAAAG	SMARCA5_qF1	GCTCCTCGACCTCCAAAAC
	SMARCA_circR1	TGTGTTGCTCCATGTCTAATCA	SMARCA5_qR1	TTGTGCCTGTGCTGCGT
RTN4	RTN4_circ1_F	TCAGGCGCCTCTTCTTAGTT	RTN4_mRNA_F	GAACGGCATCAGGCACAGATA
	RTN4_circ1_R	TGTCAATGAAAGCAGCAGGA	RTN4_mRNA_R	TCATTCAGCTTTGCGCTTCAA
DOCK1	DOCK1_Circ_F	AACCCAGAGGCACGTCCA	DOCK1_qF2	CGGCTTACACCTTGCTTCTC
	DOCK1_Circ_R	AAGAAAGTTCATCCGCTCCT	DOCK1_qR2	CTCCCACATCTTGCCTTTGT
BMPR2	BMPR2_Circ1_F	TTCCACCTCCTGACACAACA	BMPR2_mRNA_F	AGCAGTATACAGATAGGTGAGTCAA
	BMPR2_Circ1_R	GATTTCTCCCAAAGGCCATA	BMPR2_mRNA_R	CCAGCGATTCAGTGGAGATGA
CCNB1	CCNB1_circ1_F	ATGACATGGTGCACTTTCCTC	CCNB1_mRNA_F	AAGCTTGTGGCCCCTTTTA
	CCNB1_circ1_R	TAAACATGGCAGTGACACCAAC	CCNB1_mRNA_R	TCAATTGGATCCCCAGGTAA
FGD6	FGD6_Circ_F	CAAGAGTTGGAATGGCAGAA	FGD6_mRNA_F	CGGGCCTGTGGAAAGATTGTA
	FGD6_Circ_R	GCCACTGGTGGCTTCTTTAT	FGD6_mRNA_R	AGGGGAGTGCTGGTGATCTAA
TNFRSF21	TNFRSF21_Circ1_F2	ATCCCAGTGCCATTGTGGAAA	TNFRSF21_mRNA_F	AACAAGGGCTTCTTCGTGGA
	TNFRSF21_Circ1_R2	GGAATCCAAGCATGGCCATTG	TNFRSF21_mRNA_R	CACTGTGTCCTTCTTTTCTTTGGTA
GNB1	GNB1_Circ 1_F2	CAACATCGACCCAGTGGGAA	GNB1_mRNA_F	CTAACGCCAGTAGCATGTGGA
	GNB1_Circ 1_R2	ACATGTAAATTTGTCTGGAGTCTG	GNB1_mRNA_R	GGTGTCCACGTTAGTACGGTT
PVRL3	PVRL3_Circ1_F	CATCTACATTTCAGGTGCCTTAGC	PVRL3_mRNA_F	TCATCTACATTTCAGATCCTCCTAC
	PVRL3_Circ1_R	CAGTCTGTGAACTTTTGCCATGT	PVRL3_mRNA_R	TCGTGGCAATTGTGTCATCCT
SHPRH	SHPRH_Circ_F	AATGCTGAAAACTGCTGAGAGAA	SHPRH_mRNA_F	ACTTGAGGAAGAGGCCAAACA
	SHPRH_Circ_R	TTGAGAAAACGAGTGCTTTGG	SHPRH_mRNA_R	TGAATCTTTCTTTGAAGCTCATGGA
ATXN2	ATXN2_Circ_F	GGCCGAAACGTGAAGAAATA	ATXN2_mRNA_F	TACTTTGCCATTTCCACGGGC
	ATXN2_Circ_R	CATTTGGAGCCCTCTTTTTG	ATXN2_mRNA_R	TGGTGCTGATGGTGCTGAACA
ASXL1	ASXL1_circ1_F	TCTCGCATGCCTCAATGCTA	ASXL1_Ex1_qF	GGGGAGAAGGATGAAGGAC
	ASXL1_circ1_R	GTAGTTTTCTAATACCTTGAGCGTGAA	ASXL1_Ex2_qR	GTTTTGGTGTCATTGGAGCA
VEGFC	VEGFC_circF	ATCACACTTCCTGCCGATG	VEGFC_mRNA_F	CAGCAACACTACCACAGTGTC
	VEGFC_circR	ATGTTGCCAGCCTCCTTTC	VEGFC_mRNA_R	GAGTCATCTCCAGCATCCGAG
SETD3	SETD3_Circ1_F	AACACAGCTCGACAGTACGC	SETD3_Circ1_F	AACACAGCTCGACAGTACGC
	SETD3_Circ1_R	CAGCTCACTGGTCAGGTTCA	SETD3_mRNA_R	ACTGCCCACCTGTAGTCCTC

Other real-time PCR experiments were performed with the following Taqman assays according to the instructions of the manufacturer (Applied Biosystems): SEMA3G (Hs00928870_g1); SDC2 (Hs01081432_m1); CYP27A1 (Hs01017992_m1); IGFBP4 (Hs01057900_m1).

Antibodies used for the Western Blot analyses: The antibodies used for the presented Western blot analyses are listed in the table shown below.

**Antibodies**	**Source**	**Identifier (Cat#)**
β-catenin	BD Transduction Laboratories	610154
Vimentin	Cell Signaling Technology	5741
N-cadherin	BD Transduction Laboratories	610920
E-cadherin	BD Transduction Laboratories	610181
α-tubulin	Sigma	T5168

Cell cultures and transfections: Breast cancer cell lines were grown in *DMEM-F12* medium in the presence of 5% fetal calf serum. To obtain *circDOCK1* over-expressing cells, the cDNA coding for *circDOCK1* was cloned into the pcDNA3 plasmid, which was transfected in the *MDA-MB-157* and *MDA-MB-231* breast cancer cell-lines using FuGENE6 (Promega). Neomycin resistant cell clones were selected in the presence of G418 (*MDA-MB-157* cells = 400 µg/ml; *MDA-MB-231* cells = 500 µg/ml) for two weeks. Approximately twenty G418 resistant clones were isolated from each cell line and the levels of *circDOCK1* expression were examined by RT-PCR. At least three clones with highest amounts of *circDOCK1* RNA expression were expanded from each cell line and used for further experiments. To obtain negative control clones, the cells were transfected with void vectors and three of the G418 resistant clones were isolated and used for the determination of *circDOCK1* basal levels.

To obtain *circDOCK1* silencing, *CAMA1* cells were infected with lenti-viral particles containing 2 independent *circDOCK1* targeting sh-constructs cloned in the plasmid pINDUCER10 (Addgene, Watertown, MA), and the cellular pools obtained were selected in the presence of puromycin (1 µg/ml) for two weeks. Puromycin resistant cell pools were then divided into two groups and they were treated in the absence (as controls) or in the presence of 0.5 µg/ml of doxycycline for

four days to induce the shRNAs. Cells with reduced levels of *circDOCK1* relative to the basal conditions (controls) were expanded and used for further experiments.

circDOCK1 over-expression: To force the expression of *circDOCK1* in *MDA-MB-231* and *MDA-MB-157* cells, we amplified the *circDOCK1* cDNA and introduced it in a pcDNA3 plasmid vector. The sequence of the cDNA was verified and it is shown below.

***circDOCK1* cDNA sequence**

5'CTTTTTATAACTATGATGCCAGAGGAGCGGATGAACTTTCTTTACAGATCGGAGACACTGTGCACATCTT
AGAAACATATGAAGGGTGGTACCGAGGTTACACGTTACGAAAAAAGTCTAAGAAGGGTATATTTCCTGCT
TCATATATTCATCTTAAAGAAGCGATAGTTGAAGGAAAAGGGCAACATGAAACAGTCATCCCGGGTGACC
TCCCCCTCATCCAGGAAGTCACCACGACACTCCGAGAGTGGTCCACCATCTGGAGGCAGCTCTACGTGCA
AGATAACAGGGAGATGTTTCGAAGTGTGCGGCACATGATCTATGACCTTATTGAATGGCGATCACAAATT
CTTTCTGGAACTCTGCCTCAGGATGAACTCAAAGAACTGAAGAAGAAGGTCACAGCCAAAATTGATTATG
GAAACAGAATTCTAGATTTGGACCTGGTGGTTAGAGATGAAGATGGGAATATTTTGGATCCAGAATTAAC
TAGCACGATTAGTCTCTTCAGAGCTCATGAAATAGCTTCTAAACAAGTGGAGGAAAGGTTACAAGAGGAA
AAATCTCAAAAGCAGAACATAGATATTAACAGACAAGCCAAGTTTGCTGCAACCCCTTCTCTGGCCTTGT
TTGTGAACCTCAAAAATGTGGTTTGTAAAATAGGAGAAGATGCTGAAGTCCTCATGTCTCTATATGACCCT
GTGGAGTCCAAATTCATCAGTGAGAACTACCTGGTTCGCTGGTCCAGTTCAGGATTACCTAAAGACATAG
ACAGATTACATAATTTGCGAGCCGTGTTTACTGACCTCGGAAGCAAAGACCTGAAAAGGGAGAAAATCA
GTTTTGTCTGTCAGATTGTTCGCGTGGGTCGCATGGAGCTGAGGGACAACAACACCAGGAAACTGACCTC
GGGGTTGCGGCGACCTTTTGGAGTGGCTGTGATGGATGTAACAGATATAATAAATGGAAAAGTAGATGAT
GAAGATAAGCAGCATTTCATTCCCTTTCAGCCGGTGGCAGGGGAGAATGACTTCCTTCAGACTGTTATAA
ACAAAGTCATCGCTGCCAAAGAAGTCAACCACAAGGGGCAGGGTTTGTGGGTAACATTGAAATTACTTCC
TGGAGATATCCATCAGATCCGAAAAGAGTTTCCGCATTTAGTGGACAGGACCACAGCTGTGGCTCGAAAA
ACAGGGTTTCCGGAGATAATCATGCCTGGTGATGTTCGAAATGATATCTATGTAACATTAGTTCAAGGAG
ATTTTGATAAAGGAAGCAAAACAACAGCGAAGAACGTGGAGGTCACGGTGTCTGTGTACGATGAGGATG
GGAAACGATTAGAGCATGTGATTTTCCCGGGTGCTGGTGATGAAGCGATTTCAGAGTACAAATCTGTGAT
TTACTACCAAGTAAAGCAGCCACGCTGGTTTGAGACTGTTAAGGTGGCCATTCCCATCGAGGACGTTAAC
CGCAGTCACCTTCGGTTTACCTTCCGCCACAGGTCATCACAGGACTCTAAGGATAAATCTGAGAAAATAT
TTGCACTAGCATTTGTCAAGCTGATGAGATACGATGGTACCACCCTGCGAGACGGAGAGCACGATCTTAT
CGTCTATAAGGCCGAAGCAAAGAAGCTGGAAGATGCTGCCACGTACTTGAGTCTGCCCTCCACGAAGGCA
GAGTTGGAAGAAAAGGGCCACTCGGCCACCGGCAAGAGCATGCAGAGCCTTGGGAGCTGCACCATTAGC
AAGGACTCCTTCCAGATCTCCACGCTCGTGTGCTCCACCAAACTGACTCAGAACGTGGACCTTCTGGGGCT
CTTGAAATGGCGCTCCAACACCAGCCTGCTGCAGCAGAACTTGAGGCAGCTGATGAAAGTCGATGGTGGT
GAAGTAGTGAAGTTTCTTCAGGACACGTTGGATGCCCTCTTCAACATCATGATGGAGAACTCAGAGAGTG
AGACTTTTGACACGTTAGTCTTTGATGCTCTGGTATTTATCATTGGACTGATTGCTGATAGAAAATTTCAG
CATTTTAATCCTGTTTTGGAAACTTACATTAAGAAACACTTTAGTGCAACGTTAGCCTACACGAAGTTGAC
AAAAGTGTTGAAGAACTACGTGGACGGTGCTGAGAAGCCGGGAGTAAATGAGCAGCTGTACAAAGCCAT
GAAAGCGCTAGAATCCATCTTCAAGTTCATCGTGCGCTCCAGGATCCTGTTCAATCAACTGTATGAAAAC
AAGGGAGAGGCTGACTTCGTGGAATCTTTGCTGCAGCTCTTCAGGTCCATCAATGACATGATGAGCAGCA
TGTCAGACCAGACCGTCCGGGTGAAGGGGGCAGCACTGAAATACTTACCAACGATCGTCAACGATGTGA
AATTGGTGTTTGATCCCAAAGAGCTCAGCAAAATGTTTACTGAATTCATCCTCAATGTTCCCATGGGCTTG
CTGACCATCCAGAAACTCTACTGCTTGATCGAAATCGTCCACAGTGACCTCTTCACACAGCATGACTGCA
GAGAGATCCTGCTTCCCATGATGACCGATCAGCTCAAGTACCATCTGGAGAGACAGGAGGACCTGGAGG
CCTGCTGTCAGCTGCTCAGCCACATCCTGGAGGTGCTGTACAGGAAGGACGTGGGGCCAACCCAGAGGCA
CGTCCAGATTATCATGGAGAAACTTCTCCGGACCGTGAACCGAACCGTCATTTCCATGGGACGAGATTCT
GAACTCATT**3'**

The cloned circDOCK1 cDNA was introduced into the *circR* expression plasmid shown below:

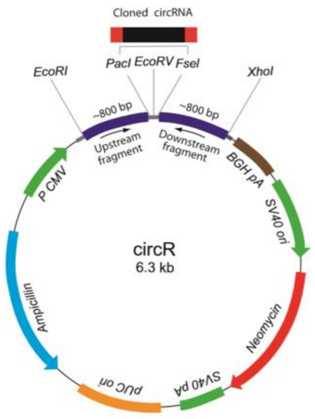

The schematic structure of the plasmid used for the over-expression of *circDOCK1* in *MDA-MB-231* and *MDA-MB-157* cells shows the site (cloned circRNA; black and red) where the *circDOCK1* cDNA was introduced.

circDOCK1 silencing studies: As for the silencing experiments, the structure of the 3 shRNAs targeting *circDOCK1* are shown below:

Sequences of the shRNAs targeting *circDOCK1*

1. ***circDOCK1-sh1*** 5'-CGAGATTCTGAACTCATTCTTT-3'

Forward:
5'TCGAGAAGGTATATTGCTGTTGACAGTGAGCGAGAGATTCTGAACTCATTCTTTTAGTGAAGCCACAGATGTAAAAGAATGAGTTC
AGAATCTCGTGCCTACTGCCTCGA**3'**
Reverse:
3'CTTCCATATAACGACAACTGTCACTCGCTCTCTAAGACTTGAGTAAGAAAATCACTTCGGTGTCTACATTTTCTTACTCAAGTCTTA
GAGCACGGATGACGGAGCTGCGC**5'**

2. ***circDOCK1-sh2*** 5'-TTCTGAACTCATTCTTTTTATA-3'

Forward:
5'TCGAGAAGGTATATTGCTGTTGACAGTGAGCGCTCTGAACTCATTCTTTTTATATAGTGAAGCCACAGATGTATATAAAAAGAATGA
GTTCAGAATGCCTACTGCCTCGA**3'**
Reverse:
3'CTTCCATATAACGACAACTGTCACTCGCGAGACTTGAGTAAGAAAAATATATCACTTCGGTGTCTACATATATTTTTCTTACTCAAG
TCTTACGGATGACGGAGCTGCGC**5'**

3. ***circDOCK1-sh3*** 5'-GAACTCATTCTTTTTATAACTA-3'

Forward:
5'TCGAGAAGGTATATTGCTGTTGACAGTGAGCGAAACTCATTCTTTTTATAACTATAGTGAAGCCACAGATGTATAGTTATAAAAAGA
ATGAGTTCTGCCTACTGCCTCGA**3'**
Reverse:

3'CTTCCATATAACGACAACTGTCACTCGCTTTGAGTAAGAAAAATATTGATATCACTTCGGTGTCTACATATCAATATTTTTCTTACTC

AAGACGGATGACGGAGCTGCGC**5'**

The sequences corresponding to the shRNAs are marked in green. The shRNAs were introduced into the pINDUCER10 lentiviral vector whose structure is shown below:

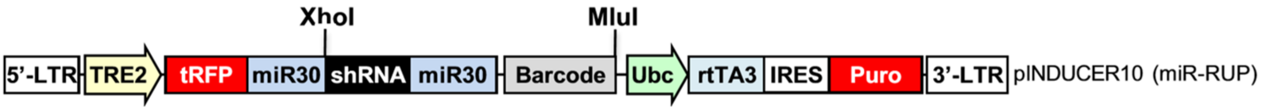

The constructs were used to infect *CAMA1* cells and silence *circDOCK1*.

Gene symbols: The list of the genes whose gene-symbols were used throughout the text is illustrated below.

ACADL	Acyl-CoA Dehydrogenase Long Chain
ADAMTSL3	ADAMTS Like 3
ADAMTS9	ADAM Metallopeptidase With Thrombospondin Type 1 Motif 9
ADGRL2	Adhesion G Protein-Coupled Receptor L2
ADPRHL1	ADP-Ribosylhydrolase Like 1
AGR2	Anterior Gradient 2, Protein Disulphide Isomerase Family Member
ALDH1A3	Aldehyde Dehydrogenase 1 Family Member A3
ANOS1	Anosmin 1
AOX1	Aldehyde Oxidase 1
APOE	Apolipoprotein E
AQP3	Aquaporin-3
ARAP2	ArfGAP with RhoGAP domain, Ankyrin Repeat and PH Domain 2
ARFGEF3	ARFGEF Family Member 3
ARHGAP22	Rho GTPase Activating Protein 22
ARHGAP31	Rho GTPase Activating Protein 31
ASPH	Aspartate Beta-Hydroxylase
ATP9B	ATPase Phospholipid Transporting 9B (Putative)
B4GALT5	Beta-1,4-Galactosyltransferase 5
BEND4	BEN Domain Containing 4
BMP4	Bone Morphogenetic Protein 4
CACNA2D4	Calcium Voltage-Gated Channel Auxiliary Subunit Alpha2delta 4
CAP2	Cyclase Associated Actin Cytoskeleton Regulatory Protein 2
CCDC77	Coiled-Coil Domain Containing 77
CD99L2	CD99 Molecule Like 2
CDC42EP4	CDC42 Effector Protein 4
CHRDL1	Chordin Like 1
CLDN4	Claudin 4
CLDN12	Claudin 12
COA1	Cytochrome C Oxidase Assembly Factor 1 Homolog
COCH	Cochlin
CRISPLD2	Cysteine Rich Secretory Protein LCCL Domain Containing 2
CSNK2A1	Casein Kinase 2 Alpha 1
CYP27A1	Cytochrome P450 Family 27 Subfamily A Member 1

DMKN	Dermokine
EHBP1	EH Domain Binding Protein 1
ENPP1	Ectonucleotiede-Pyrophosphatase/Phosphodiesterase 1
EPHB3	EPH receptor B3
FAM83A	Family With Sequence Similarity 83 Member A
FASTK	Fas Activated Serine/Threonine Kinase
FGD5-AS1	FGD5 Antisense RNA
FZD3	Frizzled Class Receptor 3
HAS2	Hyaluronan Synthase 2
GALNT1	Polypeptide N-Acetylgalactosaminyltransferase 1
GFRA1	GDNF Family Receptor Alpha 1
HEMK1	HemK Methyltransferase Family Member 1
HSPA12A	Heat Shock Protein Family A (Hsp70) Member 12A
IDS	Iduronate 2-Sulfatase
IGFN1	Immunoglobulin Like And Fibronectin Type III Domain Containing 1
IGFBP4	Insulin Like Growth Factor Binding Protein 4
IGSF3	Immunoglobulin Superfamily Member 3
ILDR2	Immunoglobulin Like Domain Containing Receptor 2
ILRAPL1	Interleukin 1 Receptor Accessory Protein
INPP5B	Inositol Polyphosphate-5-Phosphatase B
IRAK2	Interleukin 1 Receptor Associated Kinase 2
IRF5	Interferon Regulatory Factor 5
KANSL3	KAT8 Regulatory NSL Complex Subunit 3
KCNH1	Potassium Voltage-Gated Channel Subfamily H Member 1
KDM5C	lysine demethylase 5C
KRT7	Keratin 7
KRT81	Keratin 81
LINC00664	Long Intergenic Non-Protein Coding RNA 664
MBNL3	Muscleblind Like Splicing Regulator
MMD	Monocyte To Macrophage Differentiation Associated
MNX-AS1	MNX1 antisense RNA 1
MPP5	MAGUK p55 subfamily member 5
MRPS6	Mitochondrial Ribosomal Protein S6
MSRB3	Methionine Sulfoxide Reductase B3
NEK4	NIMA Related Kinase 4
NLGN1	Neuroligin 1
NRXN3	Neurexin 3
PDE10A	Phosphodiesterase 10A
PDGFD	Platelet Derived Growth Factor D
PDLIM1	PDZ And LIM Domain 1
PDZD2	PDZ Domain Containing 2
PLCD3	Phospholipase C Delta 3
PLPP3	Phospholipid Phosphatase 3
PPP1R9A	Protein-Phosphatase-1 regulatory subunit-9A
PRSS23	Serine Protease 23
PTCD3	Pentatricopeptide Repeat Domain 3
RAB11FIP2	RAB11 Family Interacting Protein 2
RAC3	Rac Family Small GTPase 3
RASSF2	Ras Association Domain Family Member 2
RBM47	RNA Binding Motif Protein 47
RETREG1	Reticulophagy Regulator 1

RHOF	Ras Homolog Family Member F, Filopodia Associated
SCD5	Stearoyl-CoA Desaturase 5
SDC2	Syndecan 2
SEMA3G	Semaphorin 3G
SEMA4C	Semaphorin 4C
SEMA5A	Semaphorin 5A
SERINC2	Serine Incorporator 2
SLC15A3	Solute Carrier Family 15 Member 3
SLC16A2	Solute Carrier Family 16 Member 2
SLITRK4	SLIT And NTRK Like Family Member 4
SNAI2	Snail Family Transcriptional Repressor 2
SNAI3	Snail Family Transcriptional Repressor 3
SNX18	Sorting Nexin 18
SP140L	SP140 Nuclear Body Protein Like
SPOCK1	SPARC (Osteonectin), Cwcv And Kazal Like Domains Proteoglycan 1
SPTLC3	Serine Palmitoyltransferase Long Chain Base Subunit 3
SRGN	Serglycin
STK25	Serine/Threonine Kinase 25
SYT2	Synaptotagmin
SYTL2	Synaptotagmin Like 2
TENM2	Teneurin Transmembrane Protein 2
TNFRSF9	TNF Receptor Superfamily Member 9
TSPAN33	Tetraspanin 33
TSR2	TSR2 Ribosome Maturation Factor
ZEB1	Zinc Finger E-Box Binding Homeobox 1
ZEB2	Zinc finger E-box-binding homeobox 2
ZNF708	Zinc Finger Protein 708

LEGENDS TO SUPPLEMENTARY TABLES

Supplementary Table S1 *RNA-seq analysis of the breast cancer cell-lines grown under basal conditions* The 20 cell-lines shown in Suppl.Fig.S1 were cultured in basal conditions and subjected to *RNA-seq* analysis. The expression levels of all the identified RNAs are shown for each of the logarithmically growing cell-line. The results obtained were used for the PCA analysis presented in Suppl.Fig.S1.

Supplementary Table S2 *List and characteristics of the 126 circular RNAs which can be generated from the DOCK1 gene* The table contains all the information available on the 126 circRNAs deriving from the *DOCK1* gene on the basis of the annotations available in the circBase website (http://www.circbase.org/). The cited circular-RNA studies are:

1) Salzman, J.; Chen R.E.; Olsen, M.N.; Wang, P.L.; Brown, P.O. Cell-type specific features of circular RNA expression. *PLoS Genet*. **2013**, *9* e1003777, doi: 10.1371/journal.pgen.1003777.

2) Jeck, W.R.; Sorrentino, J.A.; Wang, K.; Slevin,M.K.; Burd, C.E.; Liu, J.; Marzluff, W.F.; Sharpless, N.E. Circular RNAs are abundant, conserved, and associated with ALU repeats. *RNA* **2013**, *19*, 141-57, doi: 10.1261/rna.035667.112.

3) Maass, P.G.; Glažar, P.; Memczak, S.; Dittmar, G.; Hollfinger, I.; Schreyer, L.; Sauer, A.V.; Okan Toka, O.; Aiuti, A.; Luft, F.C.; Rajewsky, N. A map of human circular RNAs in clinically relevant tissues. *J Mol Med (Berl)* **2017**, *95,* 1179-1189, doi: 10.1007/s00109-017-1582-9.

4) Rybak-Wolf, A.; Stottmeister, C.; Glažar, P.; Jens, M.; Pino, N.; Giusti, S.; Hanan, M.; Behm, M.; Bartok, O.; Ashwal-Fluss, R.; Herzog, M.; Schreyer, L.; Papavasileiou, P.; Ivanov, A.; Öhman, M.; Refojo, D.; Kadener, S.; Rajewsky, N. Circular RNAs in the Mammalian Brain Are Highly Abundant, Conserved, and Dynamically Expressed. *Mol. Cell* **2015**, *58*, 870-885, doi: 10.1016/j.molcel.2015.03.027.

The list of annotations corresponding to *circDOCK1-1* (*hsa_circ_0020397*) are marked in red.

Supplementary Table S3 *RNA-seq analysis of the perturbations afforded by forced expression of circDOCK1 in the MDA-MB-231 and MDA-MB-157 breast cancer cell-lines* Three independent cell clones deriving from *MDA-MB-231* and *MDA-MB-157* cells transfected with the void plasmid vector (*pVOID1*, *pVOID2* and *pVOID3*) or the *circDOCK1* expressing plasmid (*circDOCK1a*, *circDOCK1b* and *circDOCK1c*) were subjected to *RNA-seq* analysis. The results of this analysis are summarized. Supplementary Table S2 consists of 9 sections. Each section is present in a separate sheet of the Excel file: 1) The first sheet (MDA-MB-231) contains the expression levels of all the genes identified in the *MDA-MB-231* cell clones; 2) The second sheet (MDA-MB-231_DOWN) contains the expression levels of all the genes which are significantly down-regulated in the *circDOCK1a*, *circDOCK1b* and *circDOCK1c* cell clones deriving from the *MDA-MB-231* cell-line relative to the *pVOID1*, *pVOID2* and *pVOID3* counterparts; 3) The third sheet (MDA-MB-231_UP) contains the expression levels of all the genes which are significantly up-regulated in the *circDOCK1a*, *circDOCK1b* and *circDOCK1c* cell clones deriving from the *MDA-MB-231* cell-line relative to the *pVOID1*, *pVOID2* and *pVOID3* counterparts; 4) The fourth sheet (MDA-MB-157) contains the expression levels of all the genes identified in the *MDA-MB-157* cell clones; 5) The fifth sheet (MDA-MB-157_DOWN) contains the expression levels of all the genes which are significantly down-regulated in the *circDOCK1a*, *circDOCK1b* and *circDOCK1c* cell clones deriving from the *MDA-MB-157* cell-line relative to the *pVOID1*, *pVOID2* and *pVOID3* counterparts; 6) The sixth sheet (MDA-MB-157_UP) contains the expression levels of all the genes which are significantly up-regulated in the *circDOCK1a*, *circDOCK1b* and *circDOCK1c* cell clones deriving from the *MDA-MB-157* cell-line relative to the *pVOID1*, *pVOID2* and *pVOID3* counterparts; 7) The seventh sheet (Common_DOWN) contains the expression levels of all the genes which are significantly down-regulated in the *circDOCK1a*, *circDOCK1b* and *circDOCK1c* cell clones deriving from both the *MDA-MB-231* and the *MDA-MB-157* cell-lines relative to the *pVOID1*, *pVOID2* and *pVOID3* counterparts; 8) The eigth sheet (Common_UP) contains the expression levels of all the genes which

are significantly up-regulated in the *circDOCK1a*, *circDOCK1b* and *circDOCK1c* cell clones deriving from both the *MDA-MB-231* and the *MDA-MB-157* cell-lines relative to the *pVOID1*, *pVOID2* and *pVOID3* counterparts; 9) The ninth sheet (GO Networks_COMMON) contains all the GO networks which are significantly enriched in the *circDOCK1a, circDOCK1b* and *circDOCK1c* cell clones deriving from both the *MDA-MB-231* and the *MDA-MB-157* cell-lines relative to the *pVOID1*, *pVOID2* and *pVOID3* counterparts. The pathway analysis is based on the 110 genes which are significantly up- and down-regulated in the *MDA-MB-231* and the *MDA-MB-157* cell-lines.

Supplementary Table S4 *RNA-seq analysis of the perturbations afforded by circDOCK1 silencing in the CAMA1 breast cancer cell-line* Three independent cell populations deriving from *CAMA1* cells infected with the retroviral vectors *circDOCK-sh1*, *circDOCK-sh2* or *circDOCK-sh3* were treated in duplicate with vehicle or doxycycline (DOX) and subjected to *RNA-seq* analysis. The results of this analysis are summarized. Supplementary Table S3 consists of 3 sections. Each section is present in a separate sheet of the Excel file: 1) The first sheet (CAMA1_gene expression) contains the expression levels of all the genes identified in the *CAMA1* cell populations; 2) The second sheet (DOWN-genes) contains the expression levels of all the genes which are significantly down-regulated in the Dox treated relative to the vehicle treated cell populations; 3) The third sheet (UP-genes) contains the expression levels of all the genes which are significantly up-regulated in the Dox treated relative to the vehicle treated cell populations.

Supplementary Table S5 *microRNAs predicted to bind the circDOCK1-1 RNA, the AGR2, ENPP1 and the PPP1R9A mRNAs* The first sheet of the file (binding_miRNAs) contains a list of the microRNAs which are predicted to bind *circDOCK1-1* RNA, *AGR2, ENPP1* and *PPP1R9A* mRNAs using the *in silico* approach available in the mirDB website (http://mirdb.org/mirdb/custom.html).

The second sheet of the file (common_miRNAs) provides a list of the miRNAs which are common interactors of *circDOCK1-1* RNA and *AGR2, ENPP1* or *PPP1R9A* mRNAs.

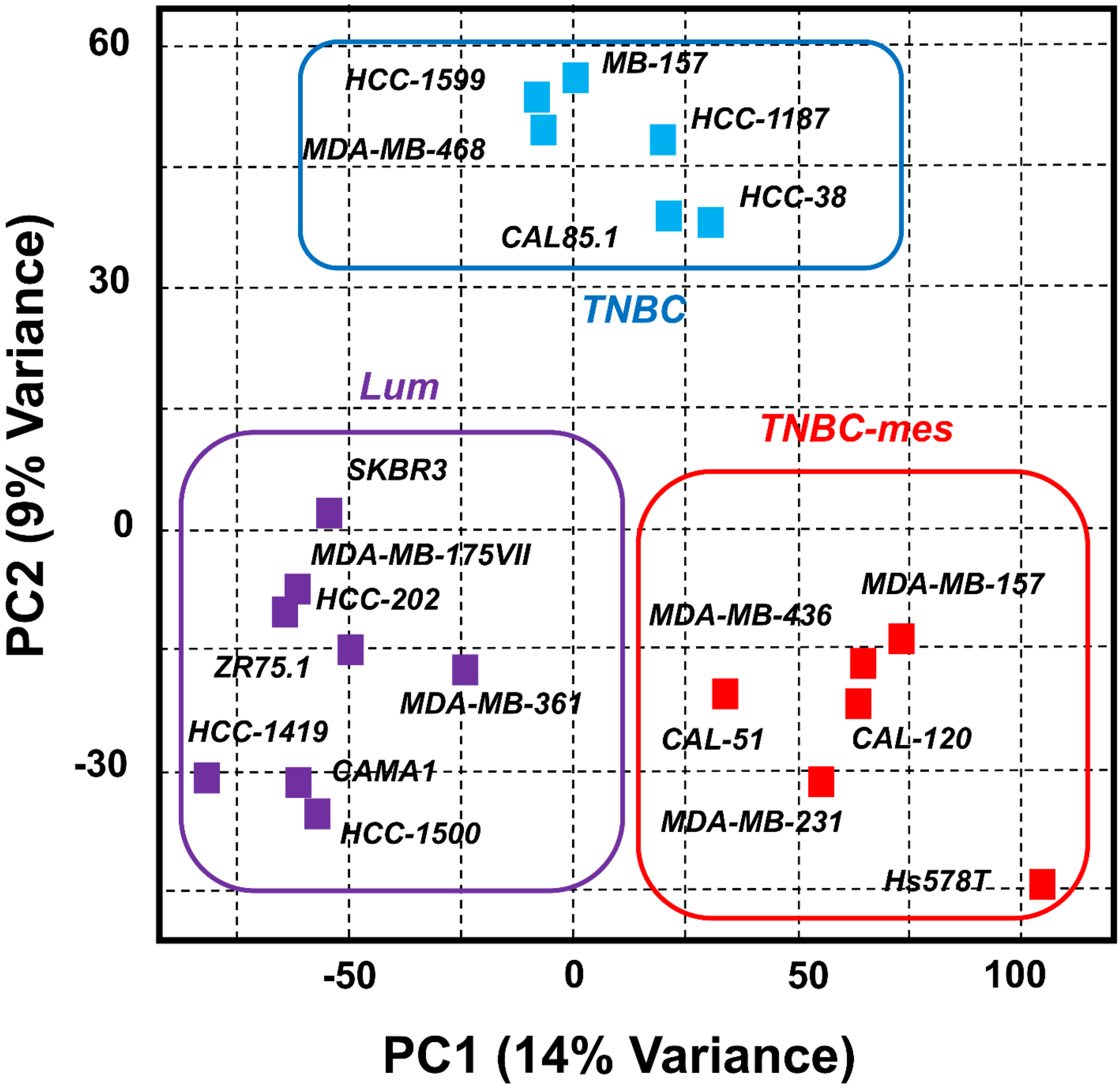

Supplementary Figure S1

Principal Component Analysis of the RNA-seq data obtained in our panel of selected breast cancer cell-lines The data demonstrate that the *RNA-seq* results separate the indicated cell-lines grown under basal conditions into three groups: Luminal (*Lum*), Triple-negative (*TNBC*) and Triple-negative with a mesenchymal morphology (*TNBC-mes*). Supplementary Table S1 contains the gene-expression data determined in each cell-line growing under basal conditions.

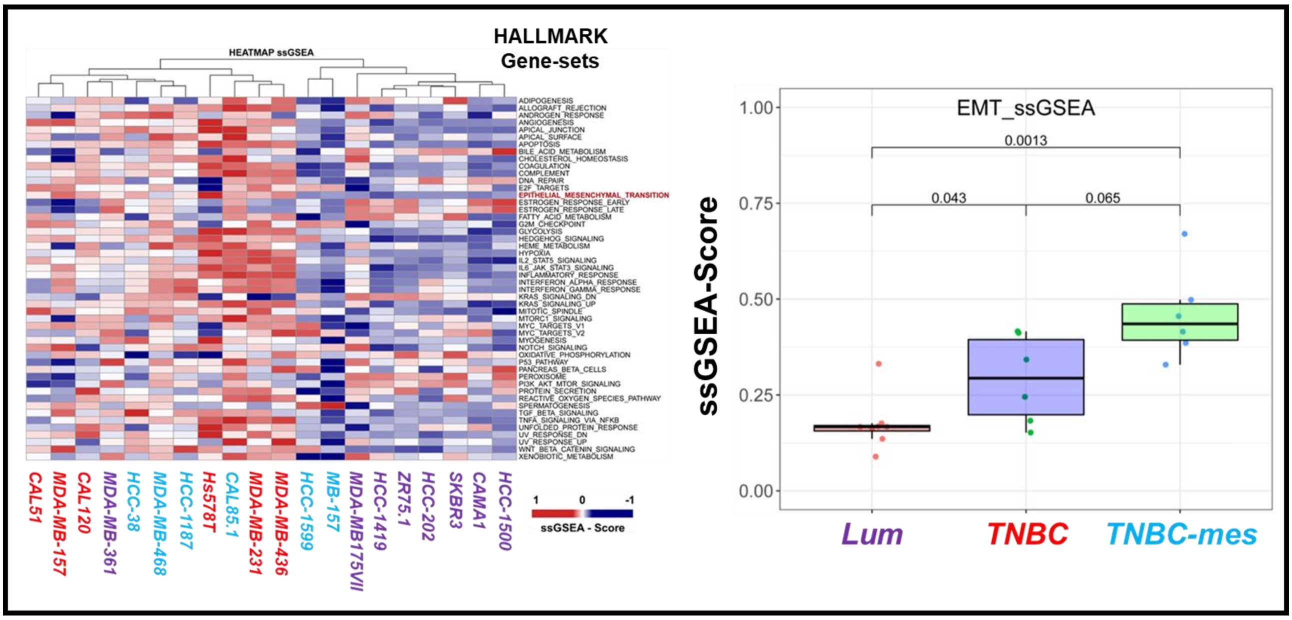

Supplementary Figure S2

Clustering of the breast cancer cell-lines according to single-sample GSEA The left panel shows the heat maps obtained following single-sample gene-set-enrichment-analysis (ssGSEA) of the *RNA-seq* data determined for each of the indicated cell-lines grown under basal conditions. The analysis is based on the 50 HALLMARKS available in the GSEA dataset. The luminal (*Lum*) cell-lines are marked in violet, the triple-negative breast-cancer (*TNBC*) cell-lines are marked in red and the *TNBC* cell-lines characterized by a mesenchymal morphology (*TNBC-mes*) are marked in blue. The epithelial-to-mesenchymal-transition (EMT) pathway is highlighted in red. The right panel shows the ssGSEA analysis performed using the EMT pathway on the three separate groups of cell-lines indicated. The p-values of the comparisons performed according to the Wilcoxon test are shown above the box-plots.

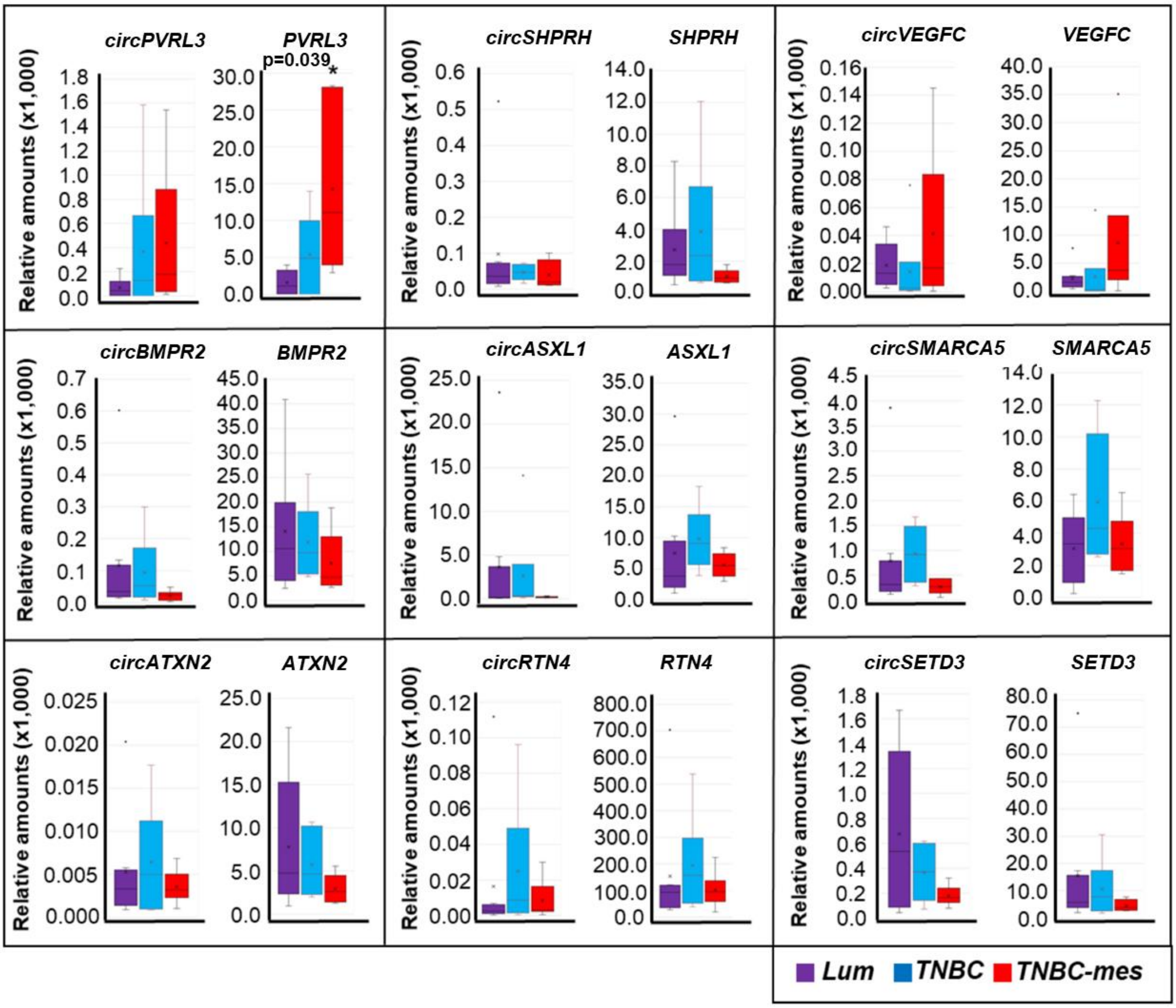

Supplementary Figure S3
Breast cancer cell-lines expression profiles of selected circRNAs up-regulated in HMLE-mes cells
The figure illustrates the box plots generated from the mean expression levels of the indicated circRNAs and the corresponding linear mRNAs which were determined in the eighteen *Lum*, *TNBC* and *TNBC-mes* cell-lines. Total RNA was extracted from logarithmically growing cell-lines cultured under standard conditions. The indicated circular RNAs and corresponding linear mRNAs were amplified with RT-PCR assays based on specific oligonucleotides and quantitated. Each value is the mean±SD of three replicate cultures of cells. *Significantly higher in the *TNBC-mes* relative to the *Lum* group, as indicated (p=0.039; Student's T-test).

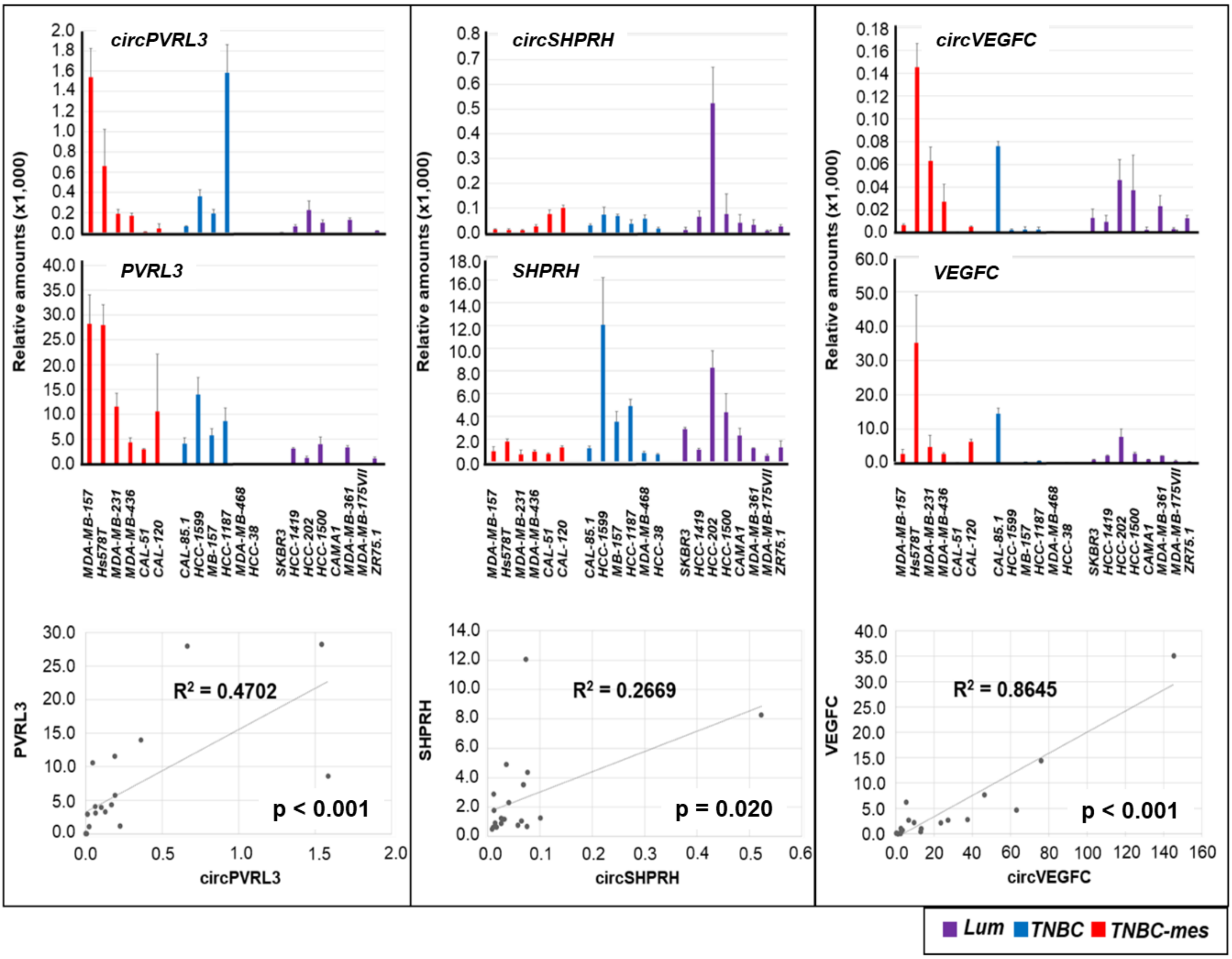

Supplementary Figure S4

Expression of circPVRL2, circSHPRH, circVEGFC circRNAs and PVRL2, SHPRH, VEGFC linear mRNAs in our panel of breast cancer cell-lines Total RNA was extracted from logarithmically growing cell-lines cultured under standard conditions. The indicated circRNAs (upper panels) and linear mRNAs (middle panels) were amplified using specific Taqman assays and quantitated. Each value is the mean±SD of three replicate cultures of cells. The lower panels illustrate the correlations between the expression of the *circPVRL2*/*circSHPRH*/*circVEGFC* RNAs and the corresponding linear mRNAs in each cell-line. The calculated R^2 correlation values (Pearson Method) and the correlation p-values are indicated.

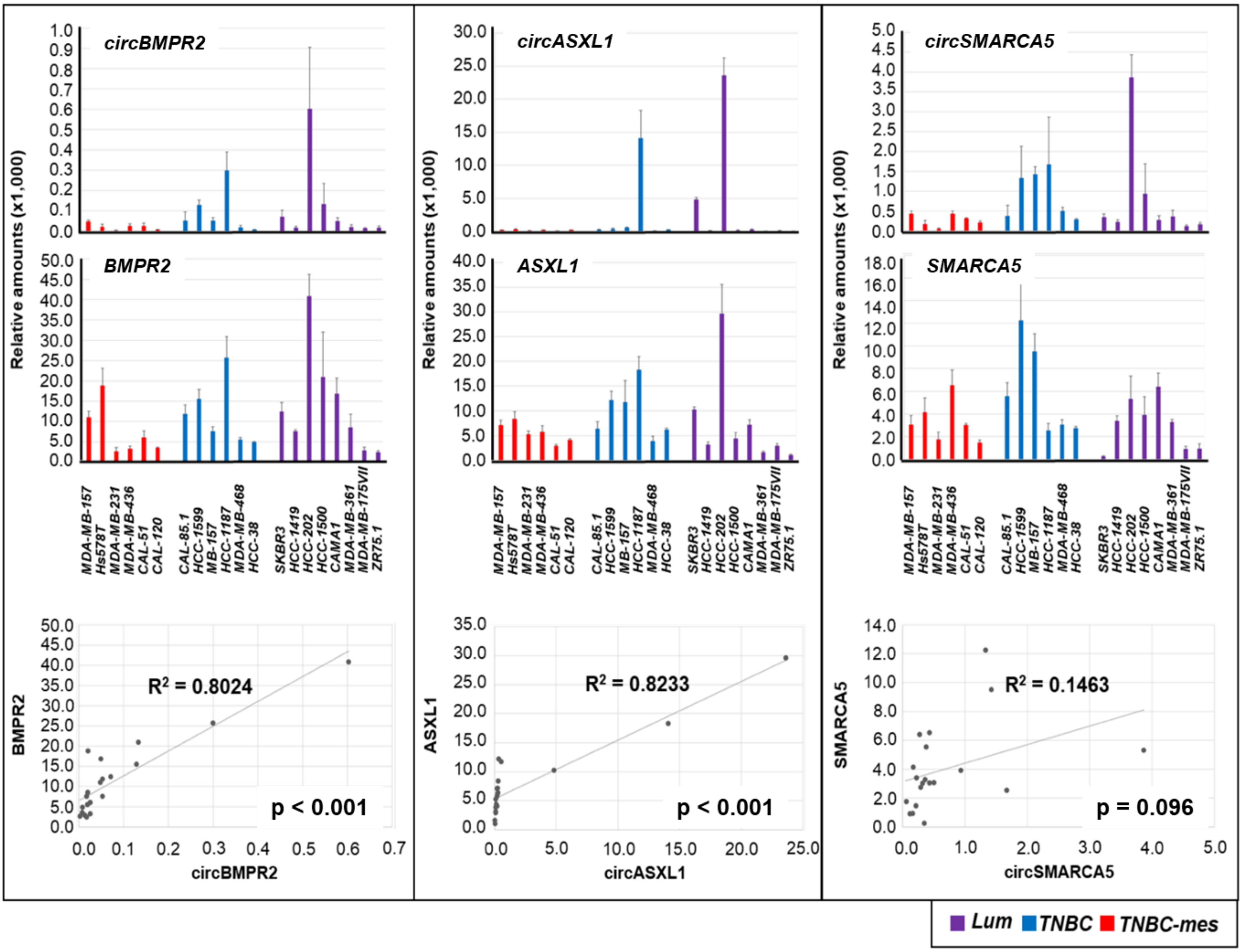

Supplementary Figure S5

Expression of circBMPR2, circASXL1, circSMARCA5 circRNAs and BMPR2, ASXL1, SMARCA5 mRNAs in our panel of breast cancer cell-lines Total RNA was extracted from logarithmically growing cell-lines cultured under standard conditions. The indicated circRNAs (upper panels) and mRNAs (middle panels) were amplified using specific Taqman assays and quantitated. Each value is the mean±SD of three replicate cultures of cells. The lower panels illustrate the correlations between the expression of *circBMPR2*/*circASXL1*/*circSMARCA5* RNAs and the corresponding linear mRNAs in each cell-line. The calculated R^2 correlation values (Pearson Method) and the correlation p-values are indicated.

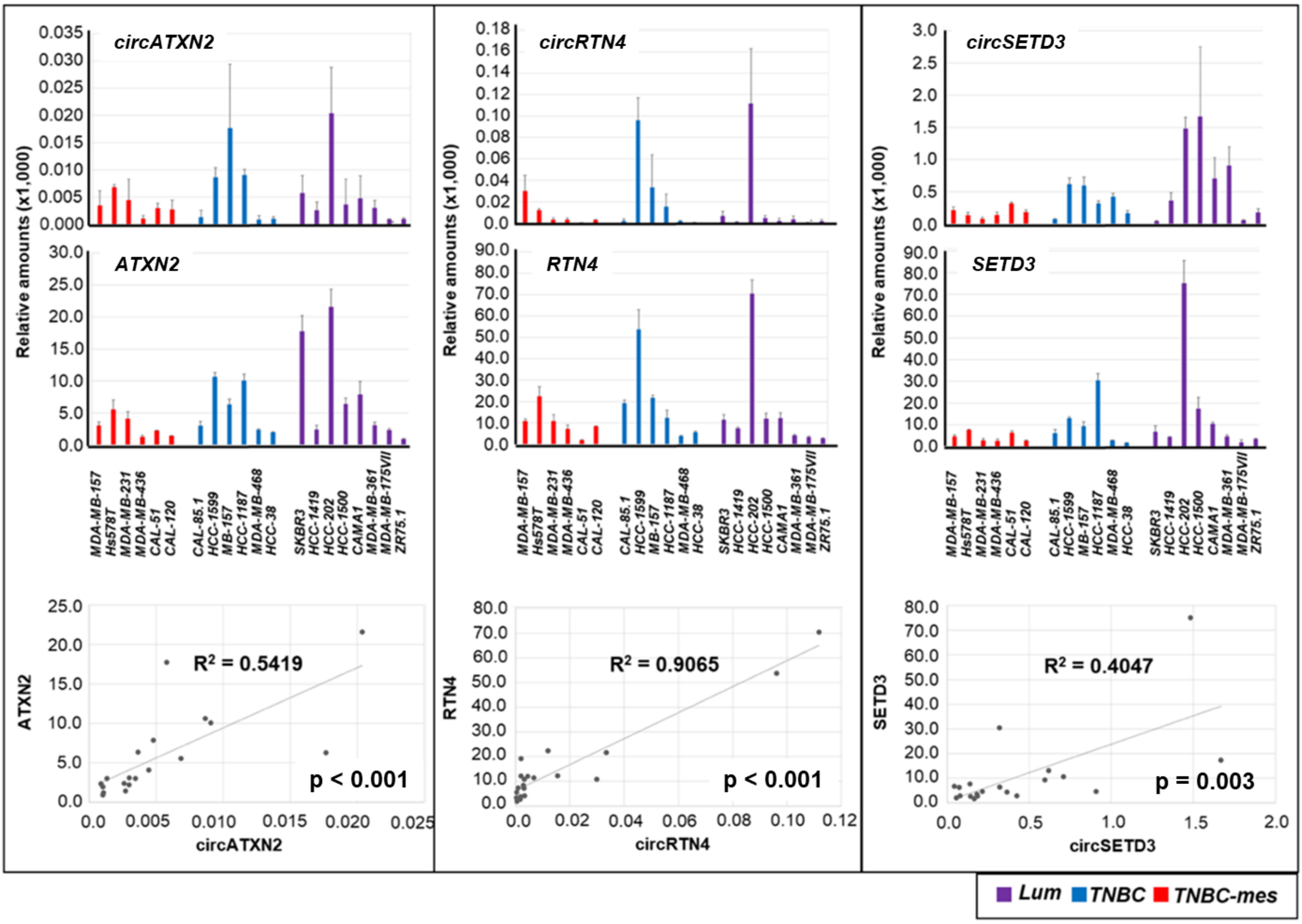

Supplementary Figure S6

Expression of circATXN2, circRTN4, circSETD3 circRNAs and ATXN2, RTN4, SETD3 mRNAs in breast cancer cell-lines Total RNA was extracted from logarithmically growing cell-lines cultured under standard conditions. The indicated circRNAs (upper panels) and mRNAs (middle panels) were amplified using specific Taqman assays and quantitated. Each value is the mean$\pm$SD of three replicate cultures of cells. The lower panels illustrate the correlations between the expression of *circATXN2*/*circRTN4*/*circSETD3* RNAs and the corresponding linear mRNAs in each cell-line. The calculated R^2 correlation values (Pearson Method) and the correlation p-values are indicated.

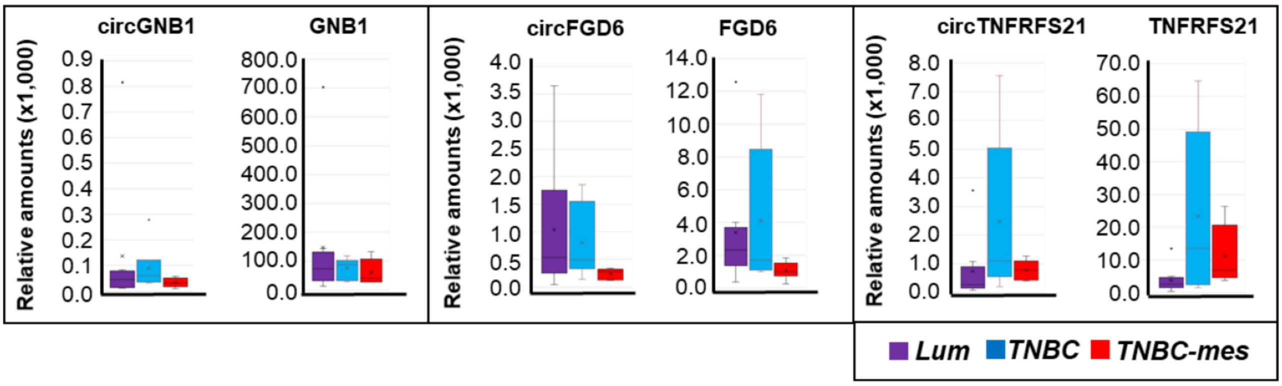

Supplementary Figure S7

Breast cancer cell-lines expression profiles of the circRNAs down-regulated in HMLE-mes cells The figure illustrates the box plots generated from the mean expression levels of the indicated circRNAs and the corresponding linear mRNAs determined in *Lum*, *TNBC* and *TNBC-mes* cell-lines. Total RNA was extracted from logarithmically growing cell-lines cultured under standard conditions. The indicated circular RNAs and corresponding linear mRNAs were amplified with RT-PCR assays based on specific oligonucleotides and quantitated. Each value is the mean±SD of three replicate cultures of cells.

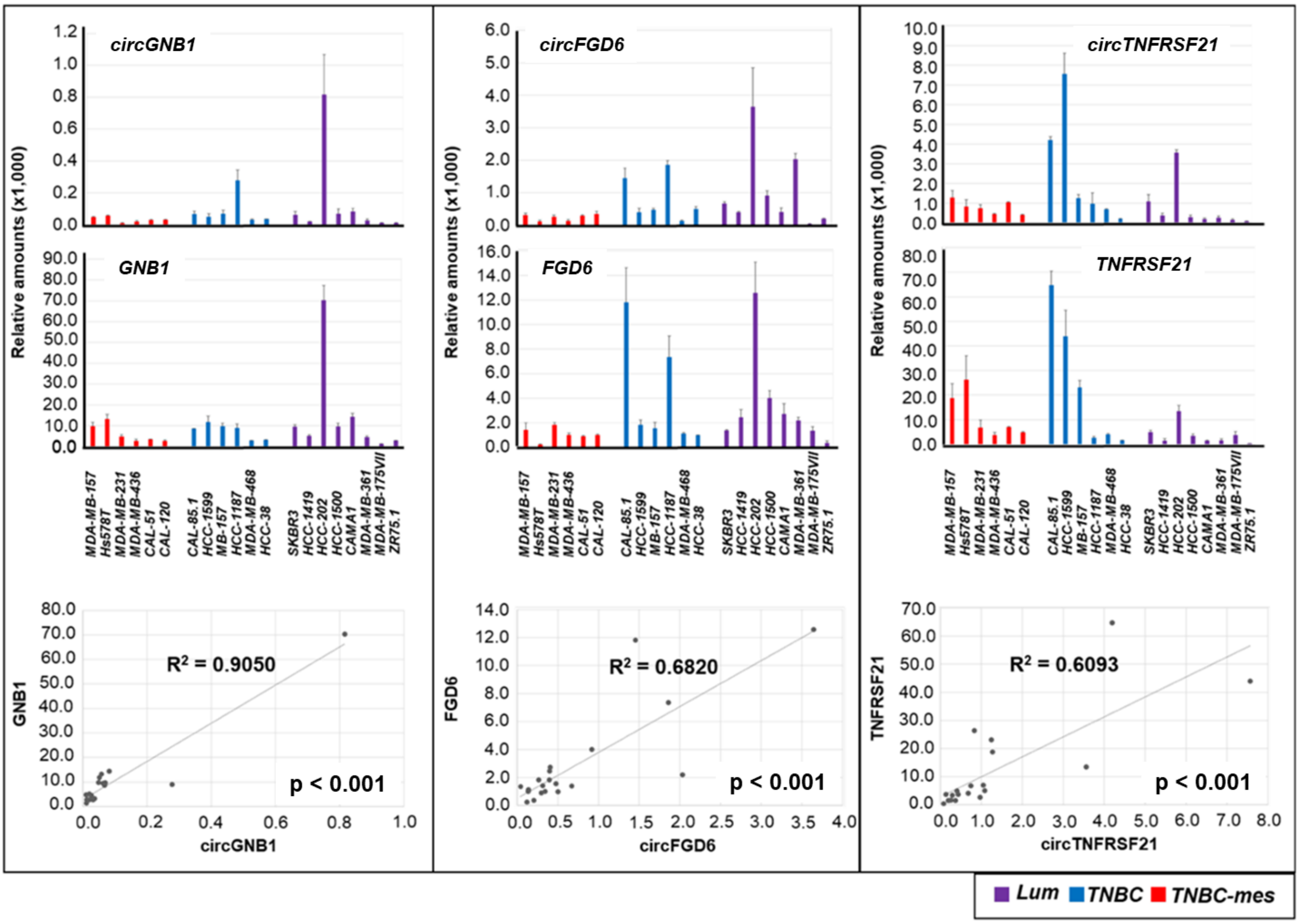

Supplementary Figure S8

Expression of circGNB1, circFGD6, circTNFRSF21 circRNAs and GNB1, FGD6, TNFRSF21 mRNAs in breast cancer cell-lines Total RNA was extracted from logarithmically growing cell-lines cultured under standard conditions. The indicated circRNAs (upper panels) and linear mRNAs (middle panels) were amplified using specific Taqman assays and quantitated. Each value is the mean±SD of three replicate cultures of cells. The lower panels illustrate the correlations between the expression of *circGNB1*/*circFGD6*/*circTNFRSF21* RNAs and the corresponding linear mRNAs in each cell-line. The calculated R^2 correlation values (Pearson Method) and the correlation p-values are indicated.

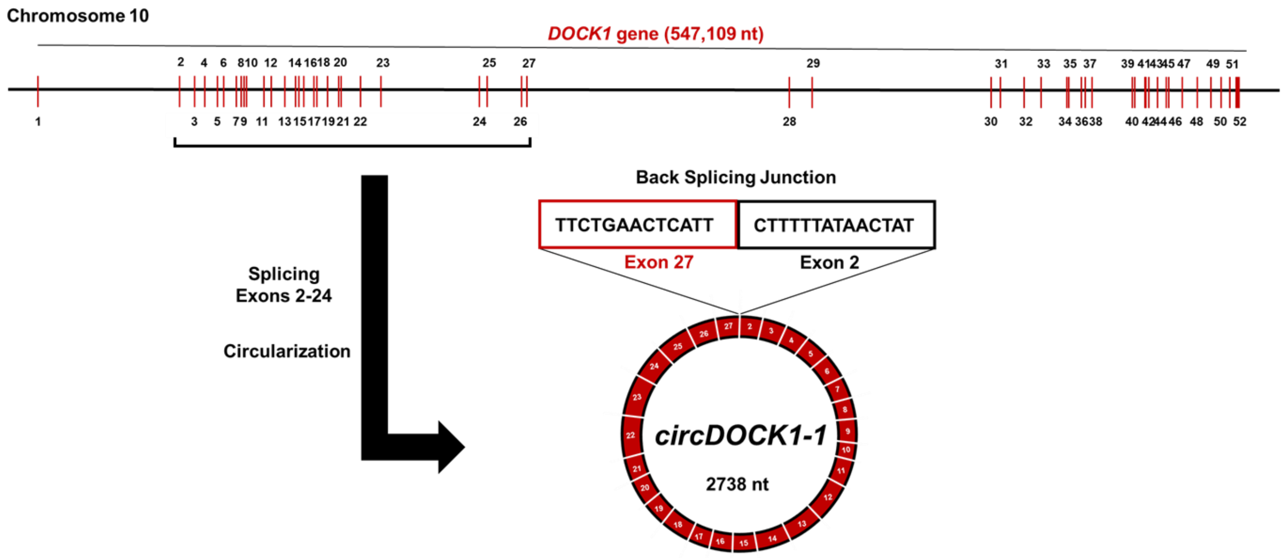

Supplementary Figure S9

Schematic representation of the structure and generation of the circDOCK1-1 RNA from the DOCK1 gene The DOCK1 gene is located on chromosome 10 and consists of 52 exons. The circDOCK1-1 RNA derives from back-splicing of exon 27 to exon 2, while the presence of the spliced exons 3-26 is assumed.

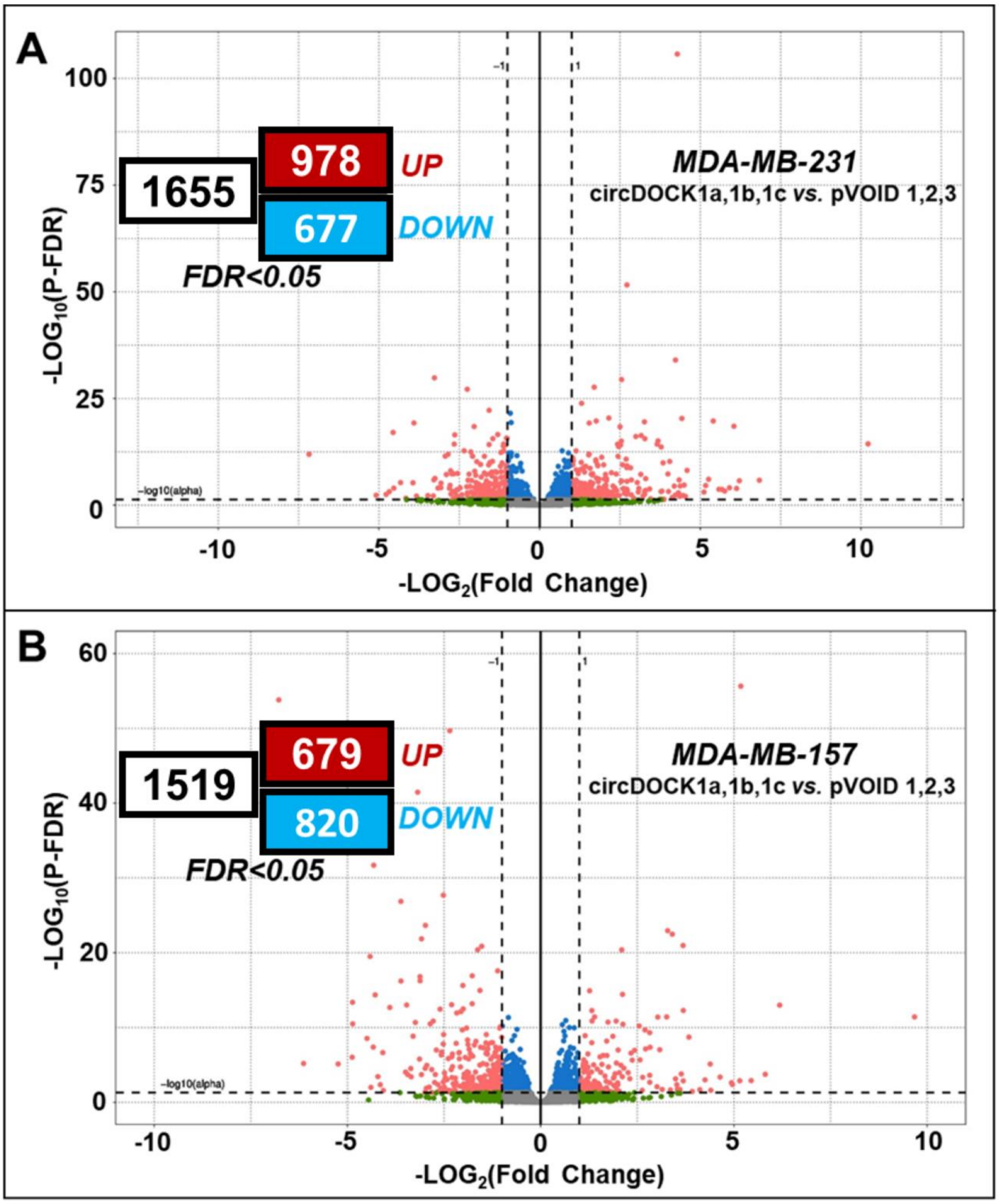

Supplementary Figure S10

Effects of circDOCK1 over-expression on the gene expression profiles of the MDA-MB231 and MDA-MB-157 cell-lines The 3 different *MDA-MB231* or *MDA-MB157* cell clones stably transfected with the *circDOCK1* containing plasmid (*circDOCK1a*; *circDOCK1b*; *circDOCK1c*) and the corresponding control *MDA-MB231* or *MDA-MB157* cell clones stably transfected with the *pVOID* empty vector (*pVOID1*; *pVOID2*; *pVOID3*) were subjected to *RNA-seq* studies. The differential gene expression profiles determined in the *circDOCK1* relative to the *pVOID* cell clones observed in the *MDA-MB231* and *MDA-MB-157* context are illustrated in panel (**A**) and panel (**B**) respectively. The data are presented as Volcano plots. The total number of differentially expressed genes is shown in the white box. The number of genes significantly up- and down-regulated [False Discovery Rate (FDR) < 0.05] is contained in the red and blue boxes, respectively.

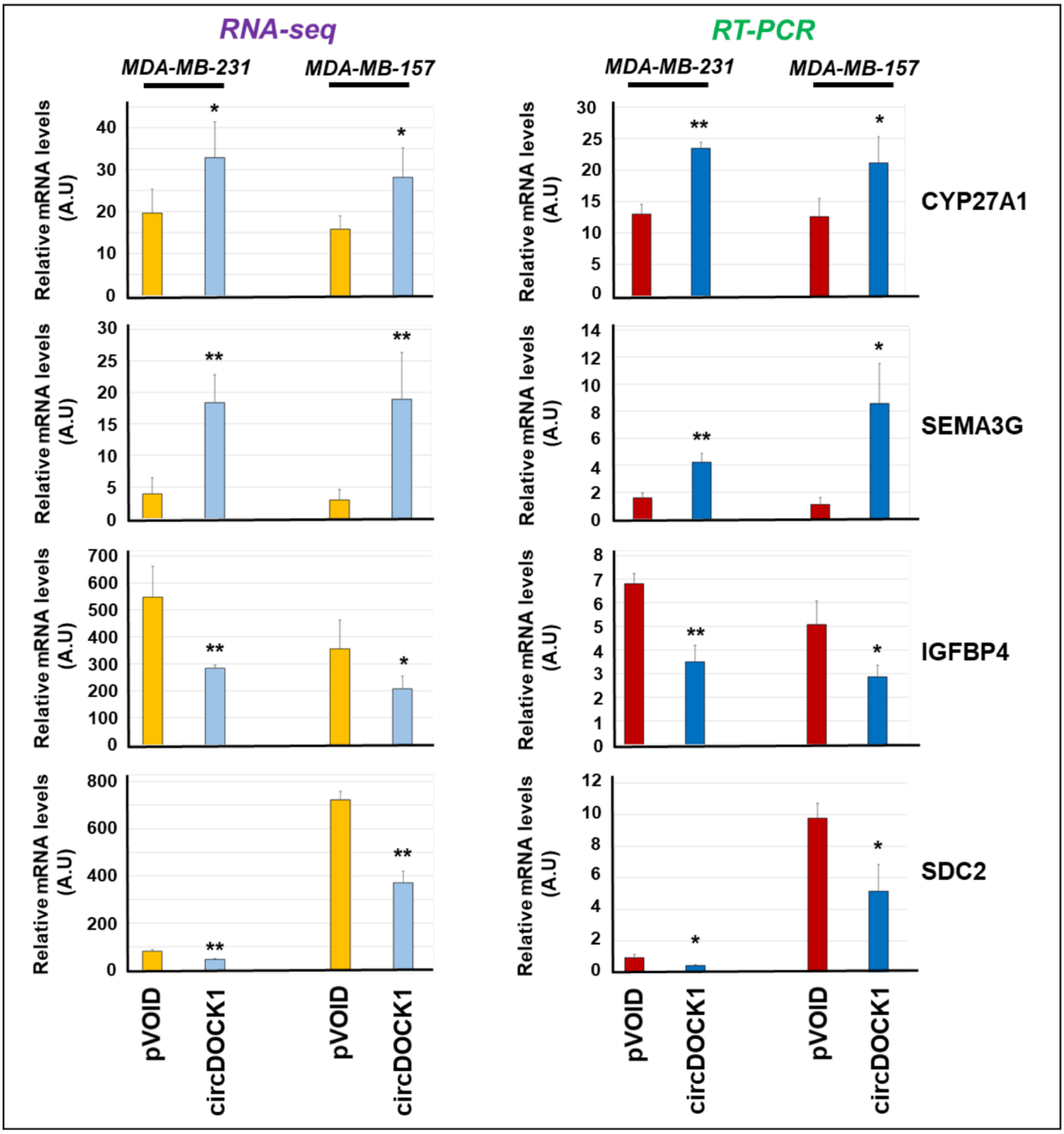

Supplementary Figure S11

Validation of the RNA-seq results on two up-regulated and two down-regulated genes by PCR analysis The left panels illustrate the expression levels of the indicated genes obtained with the *RNA-seq* studies performed in the three *MDA-MB-231* and *MDA-MB-157* cell clones stably transfected with the *circDOCK1* plasmid and the corresponding *pVOID* vector. The right panels illustrate the RT-PCR validation results obtained with the same samples used for the *RNA-seq* experiments. The PCR experiments were performed with the use of commercially available Taqman assays targeting the indicated genes. Each value is the Mean$\pm$SD of the three indicated cell clones. **Significantly different from the corresponding *pVOID* value (p<0.01 following two-tailed Student's t-test). *Significantly different from the corresponding *pVOID* value (p<0.05 following two-tailed Student's t-test).

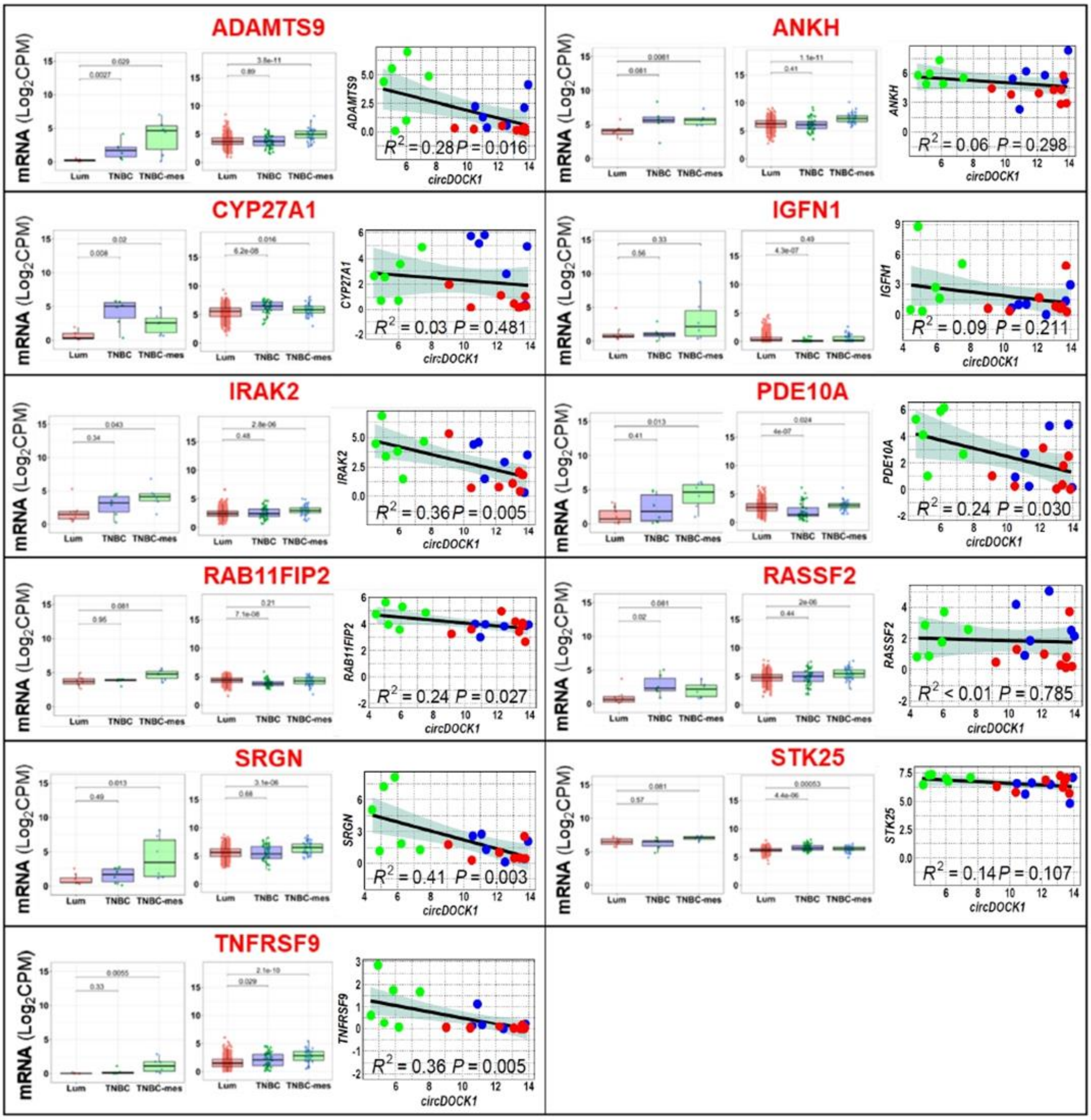

Supplementary Figure S12

Expression profiles of some of the genes up-regulated by circDOCK1-1 in breast cancer cell-lines and tumor specimens The indicated mRNAs are coded by some of the genes which are commonly up-regulated in *MDA-MB-231* and *MDA-MB-157* cells over-expressing *circDOCK1-1*, according to our *RNA-seq* data. These mRNAs are characterized by a trend towards higher expression levels in the *TNBC-mes* relative to the *Lum* cell types belonging to our panel of 18 cell-lines (left diagrams of each panel; the p-values of the indicated comparisons are shown, Student's T-test). The expression profiles of the mRNAs which can be determined from the *RNA-seq* data obtained from the breast cancer specimens available in the TCGA (The Cancer Genome Atlas) database are also illustrated (middle diagrams of each panel; the p-values of the indicated comparisons are shown, Student's T-test). The right diagrams of each panel show the quantitative correlations between the levels of the indicated mRNAs and *circDOCK1* in each breast cancer cell-line considered. *TNBC-mes*, *TNBC* and *Lum* cell-lines are marked by the green, blue and red dots, respectively. The calculated R^2 correlation value (Pearson Method) and the correlation p-value are indicated.

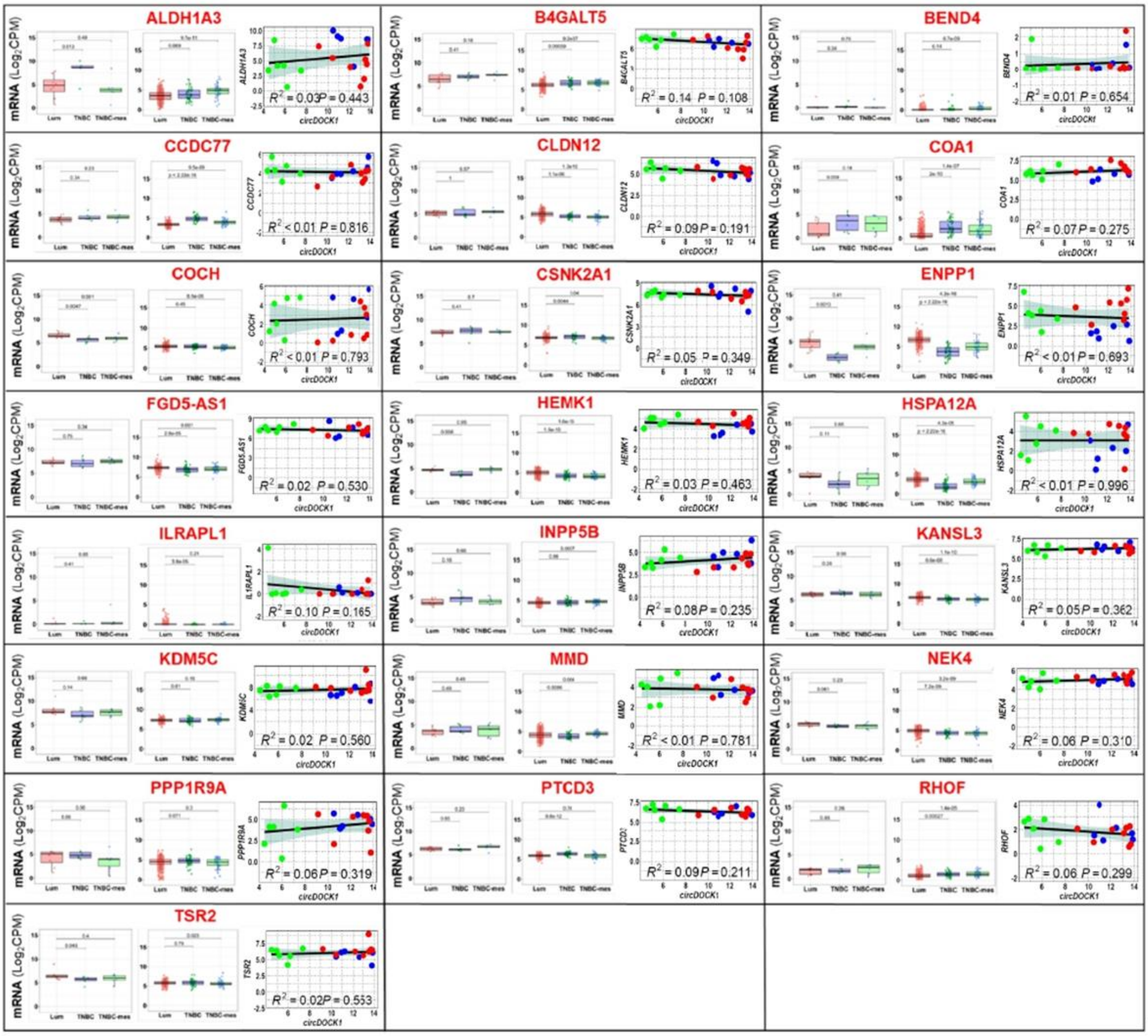

Supplementary Figure S13

Expression profiles of some of the genes up-regulated by circDOCK1-1 in breast cancer cell-lines and tumor specimens The indicated RNAs are coded by some of the genes which are commonly up-regulated in *MDA-MB-231* and *MDA-MB-157* cells over-expressing *circDOCK1*, according to our *RNA-seq* data. These RNAs are characterized by similar expression levels in the *TNBC-mes* and *Lum* cell types belonging to our panel of 18 cell-lines (left diagrams of each panel; the p-values of the indicated comparisons are shown, Student's T-test). The expression profiles of the mRNAs which can be determined from the *RNA-seq* data obtained with the breast cancer specimens available in the TCGA (The Cancer Genome Atlas) database are also illustrated (middle diagrams of each panel; the p-values of the indicated comparisons are shown, Student's T-test). The right diagrams of each panel show the quantitative correlations between the levels of the indicated mRNAs and *circDOCK1* in each breast cancer cell-line considered. *TNBC-mes*, *TNBC* and *Lum* cell-lines are marked by the green, blue and red dots, respectively. The calculated R^2 correlation value (Pearson Method) and the correlation p-value are indicated.

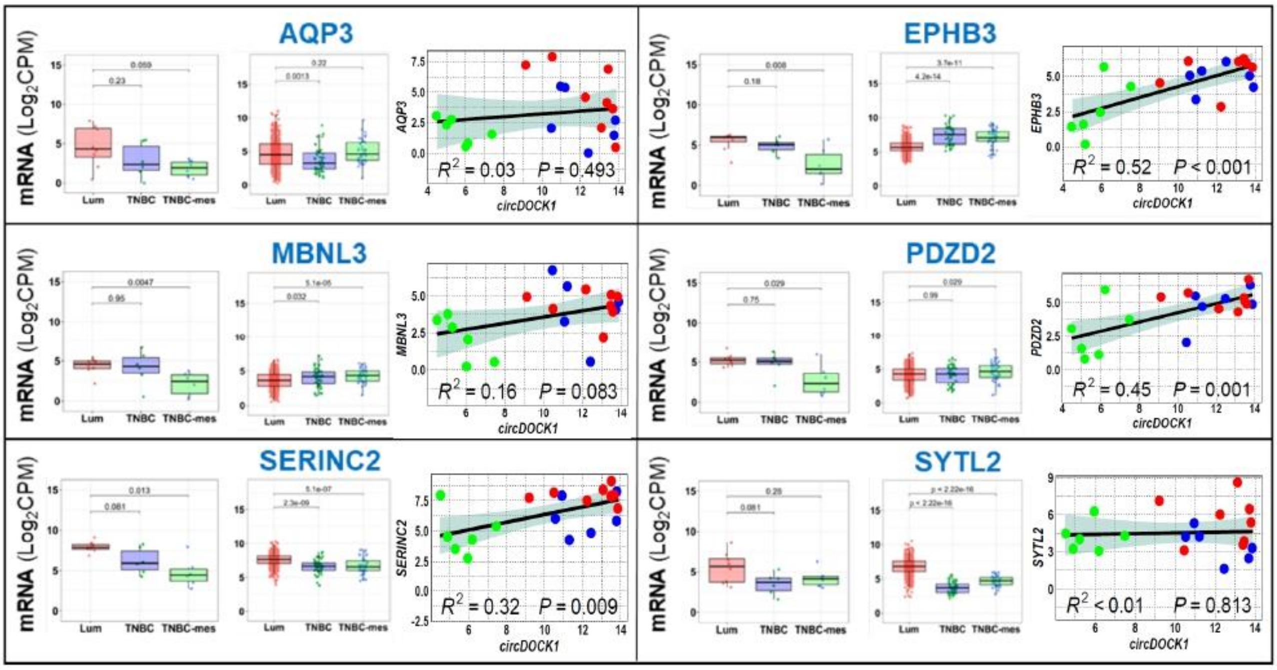

Supplementary Figure S14

Expression profiles of some of the genes down-regulated by circDOCK1in breast cancer cell-lines and tumor specimens The indicated mRNAs are coded by some of the genes which are commonly down-regulated in *MDA-MB-231* and *MDA-MB-157* cells over-expressing *circDOCK1*, according to our *RNA-seq* data. These mRNAs are characterized by a trend towards lower expression levels in the *TNBC-mes* relative to the *Lum* cell types belonging to our panel of 18 cell-lines (left diagrams of each panel; the p-values of the indicated comparisons are shown, Student's T-test). The expression profiles of the mRNAs which can be determined from the *RNA-seq* data obtained from the breast cancer specimens available in the TCGA (The Cancer Genome Atlas) database are also illustrated (middle diagrams of each panel; the p-values of the indicated comparisons are shown, Student's T-test). The right diagrams of each panel show the quantitative correlations between the levels of the indicated mRNA and *circDOCK1* in each breast cancer cell-line considered. *TNBC-mes*, *TNBC* and *Lum* cell-lines are marked by the green, blue and red dots, respectively. The calculated R^2 correlation value (Pearson Method) and the correlation p-value are indicated.

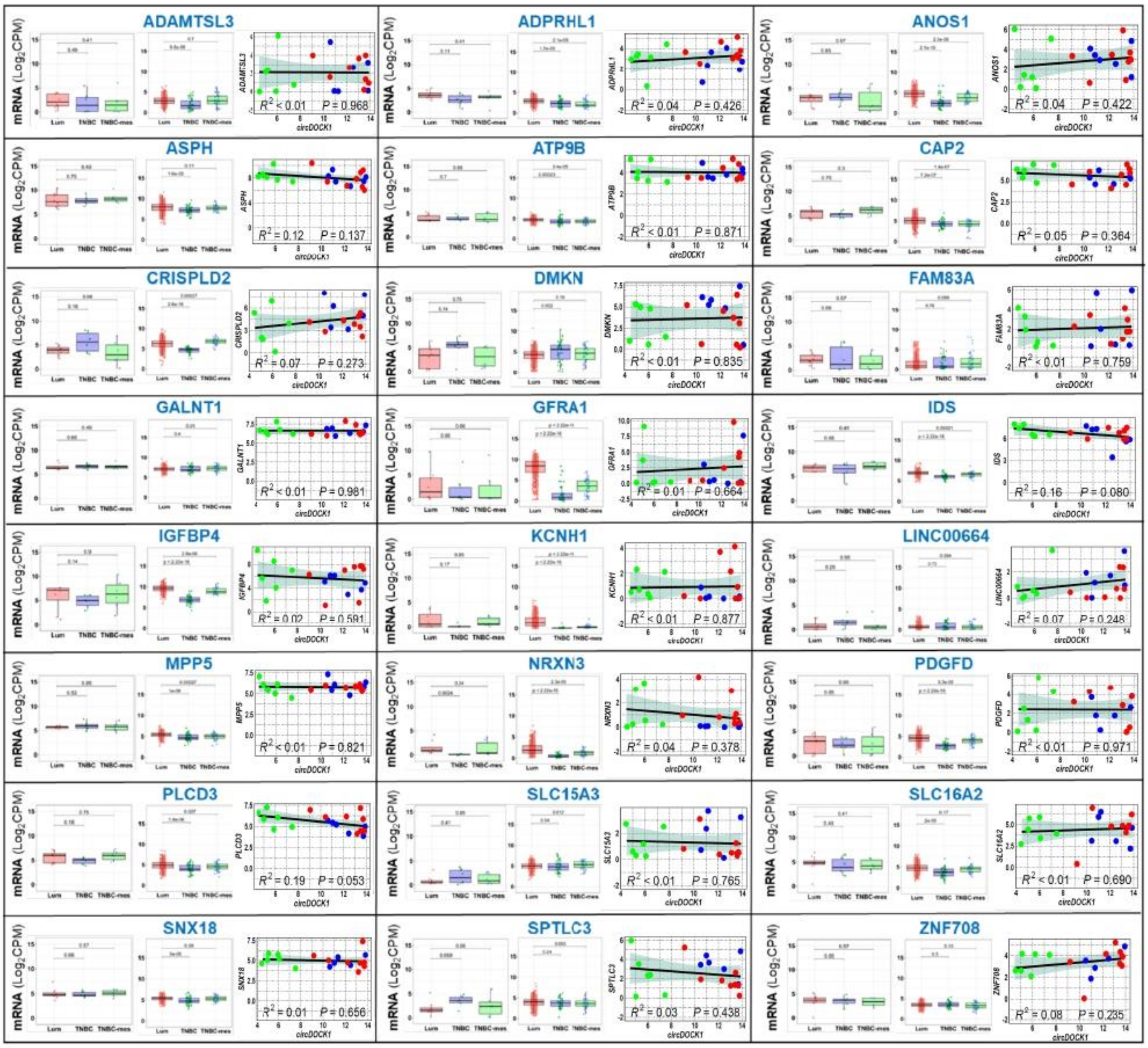

Supplementary Figure S15

Expression profiles of some of the genes down-regulated by circDOCK1-1 in breast cancer cell-lines and tumor specimens The indicated RNAs are coded by some of the genes which are commonly down-regulated in *MDA-MB-231* and *MDA-MB-157* cells over-expressing *circDOCK1-1*, according to our *RNA-seq* data. These RNAs are characterized by similar expression levels in the *TNBC-mes* and *Lum* cell-lines belonging to our panel of 18 cell-lines (left diagrams of each panel; the p-values of the indicated comparisons are shown, Student's T-test). The expression profiles of the RNAs which can be determined from the *RNA-seq* data obtained from the breast cancer specimens available in the TCGA (The Cancer Genome Atlas) database are also illustrated (middle diagrams of each panel; the p-values of the indicated comparisons are shown, Student's T-test). The right diagrams of each panel show the quantitative correlations between the levels of the indicated RNA and *circDOCK1-1* in each breast cancer cell-line considered. *TNBC-mes*, *TNBC* and *Lum* cell-lines are marked by the green, blue and red dots, respectively. The calculated R^2 correlation value (Pearson Method) and the correlation p-value are indicated.

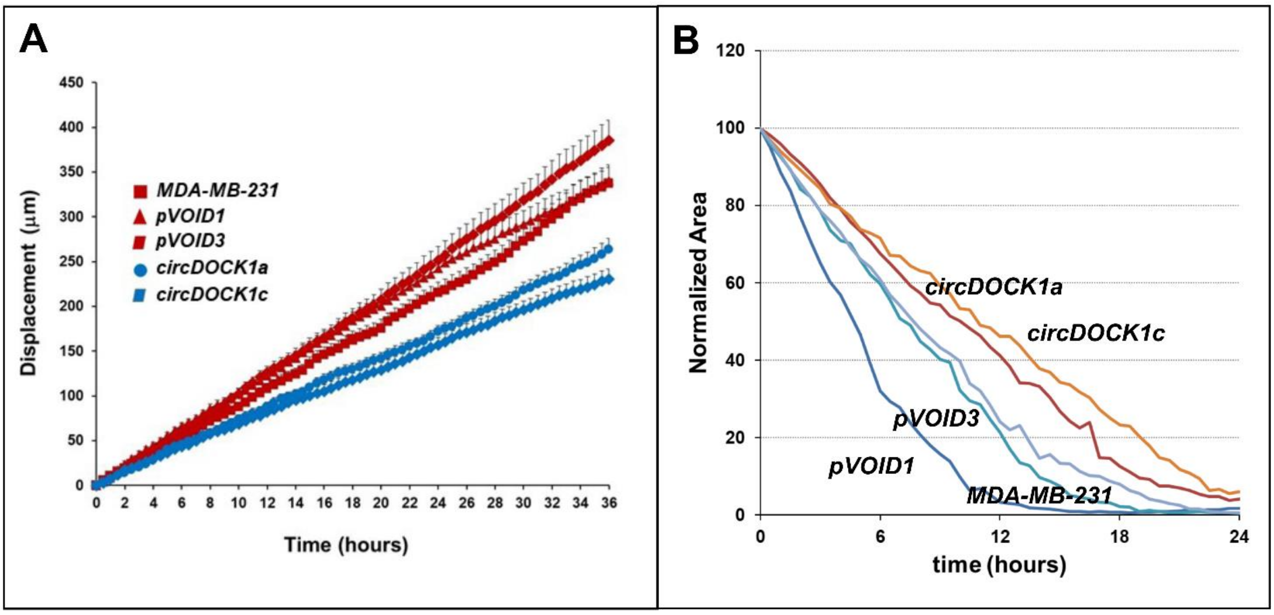

Supplementary Figure S16

Effects of circDOCK1-1 over-expression on random motility and wound-healing Parental *MDA-MB-231* as well as two derived cell clones stably transfected with a plasmid allowing the expression of *circDOCK1-1* (*circDOCK1a*; *circDOCK1c*) and the corresponding void vector (*pVOID1*; *pVOID3*) were used for the experiments described in the various panels. (**A**) The diagrams illustrate the random motility values determined in parental *MDA-MB-231*, *pVOID1*, *pVOID3*, *circDOCK1a* and *circDOCK1c* cells by time-lapse microscopy at the indicated time points. The motility curves of *circDOCK1a* and *circDOCK1c* cells are significantly lower than the corresponding curves generated from the data obtained with parental *MDA-MB-231 pVOID1* and *pVOID3* cells ($p < 0.001$ following two-way ANOVA Bonferroni post-test). (**B**) The diagrams show the wound healing curves determined in parental *MDA-MB-231*, *pVOID1*, *pVOID3*, *circDOCK1a* and *circDOCK1c* cells by time-lapse microscopy at the indicated time points. The values indicate the void area contained between the margins of the healing. The curves obtained with *circDOCK1a* and *circDOCK1c* cells indicate that the closure of the wound is slower than what is observed with parental *MDA-MB-231 pVOID1* and *pVOID3* cells ($p < 0.001$ following two-way ANOVA Bonferroni post-test).

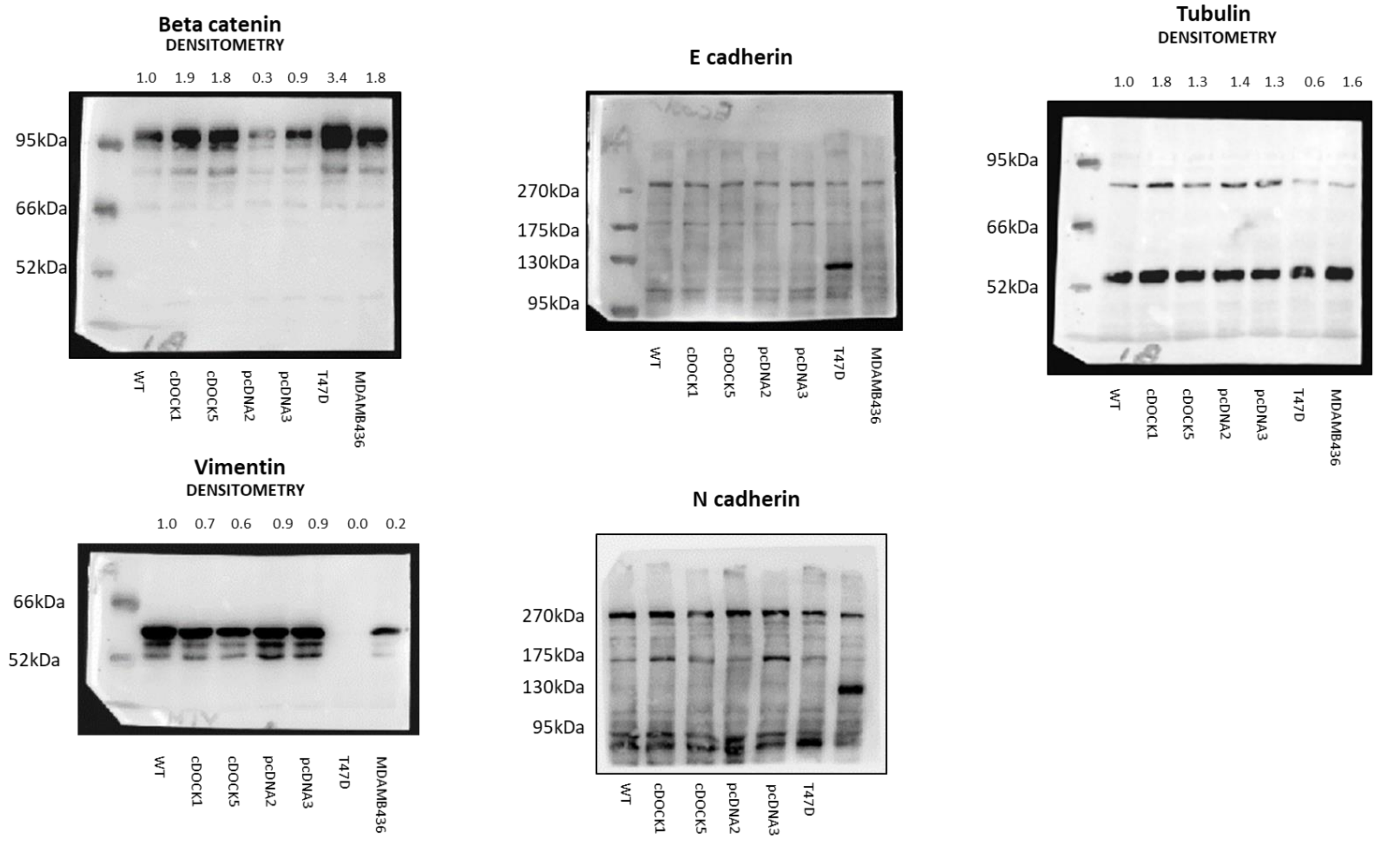

Original Western blots contained in Fig. 7C
